# Myeloid deficiency of Z‐DNA binding protein 1 restricts septic cardiomyopathy via promoting macrophage polarisation towards the M2‐subtype

**DOI:** 10.1002/ctm2.70315

**Published:** 2025-04-27

**Authors:** Yifan Shi, Lu He, Jie Ni, Yuyuan Zhou, Xiaohua Yu, Yao Du, Yang Li, Xi Tan, Yufang Li, Xiaoying Xu, Si Sun, Lina Kang, Biao Xu, Jibo Han, Lintao Wang

**Affiliations:** ^1^ Department of Cardiology Nanjing Drum Tower Hospital Affiliated Hospital of Medical School Nanjing University Nanjing Jiangsu China; ^2^ Department of Neurosurgery The First Affiliated Hospital Hengyang Medical School University of South China Hengyang Hunan China; ^3^ Department of Cardiology Nanjing Drum Tower Hospital Clinical College of Nanjing University of Chinese Medicine Nanjing Jiangsu China; ^4^ Department of Cardiology the Second Affiliated Hospital of Jiaxing University Jiaxing Zhejiang China; ^5^ Nanjing Key Laboratory for Cardiovascular Information and Health Engineering Medicine Institute of Clinical Medicine Nanjing Drum Tower Hospital Medical School Nanjing University Nanjing Jiangsu China

**Keywords:** macrophage polarisation, myocardial dysfunction, sepsis, STAT1, ZBP1

## Abstract

**Background:**

Septic cardiomyopathy is a frequent complication in patients with sepsis and is associated with a high mortality rate. Given its clinical significance, understanding the precise underlying mechanism is of great value.

**Methods and results:**

Our results unveiled that Z‐DNA binding protein 1 (ZBP1) is upregulated in myocardial tissues of lipopolysaccharide (LPS)‐treated mice. Single‐cell mRNA sequencing (scRNA‐seq) and single‐nucleus mRNA sequencing (snRNA‐seq) indicated that *Zbp1* mRNA in endothelial cells, fibroblasts and macrophages appeared to be elevated by LPS, which is partially consistent with the results of immunofluorescence. Through echocardiography, we identified that global deletion of ZBP1 improves cardiac dysfunction and the survival rate of LPS‐treated mice. Mechanistically, snRNA‐seq showed that ZBP1 is mainly expressed in macrophages and deletion of ZBP1 promotes the macrophage polarisation towards M2‐subtype, which reduces inflammatory cell infiltration. Notably, myeloid‐specific deficiency of ZBP1 also promotes M2 macrophage polarisation and improves cardiac dysfunction, validating the role of macrophage‐derived ZBP1 in septic myocardial dysfunction. Finally, we revealed that LPS increases the transcription and expression of ZBP1 through signal transducer and activator of transcription 1 (STAT1). Fludarabine, the inhibitor of STAT1, could also promote M2 macrophage polarisation and improve cardiac dysfunction of LPS‐treated mice.

**Conclusions:**

Our study provides evidence of a novel STAT1‐ZBP1 axis in macrophage promoting septic cardiomyopathy, and underscores the potential of macrophage‐derived ZBP1 as a therapeutic target for septic cardiomyopathy.

**Key points:**

- Macrophage‐derived ZBP1 exacerbates LPS‐induced myocardial dysfunction and inflammatory cell infiltration.
- Deletion of ZBP1 promotes macrophage polarisation from M1 to M2.
- STAT1‐ZBP1 axis promotes septic cardiomyopathy.
- ZBP1 has emerged as a potential therapeutic target for inflammation and septic cardiomyopathy.

## Graphical abstract

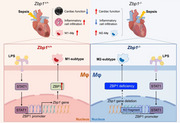

## 1 INTRODUCTION

Sepsis is a life‐threatening condition resulting from various harmful immune reactions, which may develop into systemic inflammatory response syndrome and ultimately cause multiple organ failure.^1^ Myocardial dysfunction triggered by sepsis is a severe complication that occurs in sepsis patients. Approximately 20%–60% of sepsis patients are likely to develop cardiomyopathy.^2^ Patients experiencing myocardial injury from sepsis can potentially show clinical manifestation of cardiac dysfunction and left ventricular dilation in the early, and recover in about 7–10 days.^2^ Although macrophages constitute only a modest proportion (∼10%) of the cellular components in the heart, their function has been reported to play a significant role in the pathogenesis of septic myocardial dysfunction.^3^ Among these mechanisms, macrophage polarisation is particularly crucial. During sepsis, a substantial number of macrophages infiltrate the myocardium, which adopt a pro‐inflammatory phenotype (M1 macrophages).^4^ The polarisation of M1 macrophages prolongs the inflammatory response, thereby disrupting energy metabolism, stimulating excessive nitric oxide production, unbalancing calcium homeostasis, and ultimately leading to myocardial dysfunction.^5^ Therefore, elucidating the mechanisms underlying cardiac macrophage polarisation could enhance our understanding of the pathogenesis of septic cardiomyopathy.

Z‐DNA binding protein 1 (ZBP1) was originally identified as a nucleic acid sensing protein via its Zα domain^6^ and is newly identified as a driver of inflammatory cell death through its RHIM domain.^7^ ZBP1 is also known to be involved in regulating inflammatory phenotype of macrophages.^8^ Recent studies have revealed that ZBP1 promotes the inflammasome activation and IL‐1β release in lipopolysaccharide (LPS)‐induced macrophages.^9^ Wang et al. discovered that ZBP1 was mainly upregulated in M1 macrophages compared to either M0 or M2 polarised macrophages.^10^ Further, overexpression of ZBP1 inhibited M2 macrophage polarisation, and on the contrary, knockdown of ZBP1 promotes macrophage polarisation from M1 to M2.^10^ In regard to heart disorders, the ZBP1 expression is upregulated in cardiac myocardial tissues of mice with doxorubicin‐induced cardiotoxicity and myocardial infarction.^11^, ^12^ However, whether ZBP1 and M1 macrophage polarisation it mediates participate in sepsis‐induced myocardial dysfunction remains unclear.

In this study, we aimed to investigate the effect of ZBP1 on septic cardiomyopathy and elucidate the behind underlying mechanism. Initially, our data showed that ZBP1 was upregulated in myocardial tissues of LPS‐induced mice. Global deletion of ZBP1 protected against septic myocardial dysfunction. Mechanistically, snRNA‐seq and scRNA‐seq showed that ZBP1 was mainly expressed in macrophages and alters the ratio of M1 and M2 macrophages in the hearts of LPS‐treated mice. Further pseudotime analysis revealed that deletion of ZBP1 promoted the macrophage polarisation towards M2‐subtype. Next, myeloid‐specific *Zbp1* knockout mice were generated to validate the role of macrophage‐derived ZBP1 in septic myocardial dysfunction. Finally, we explored that LPS increased the transcription and expression of ZBP1 through signal transducer and activator of transcription (STAT1). Our study underscores the potential of macrophage‐derived ZBP1 as a therapeutic target for septic cardiomyopathy and offers a novel mechanism of ZBP1 regulating macrophage activation.

## 2 MATERIALS AND METHODS

### 2.1 Mice experiments

All animal procedures were reviewed and approved by the Institutional Ethics Committee of Nanjing Drum Tower Hospital. Experiments were conducted in strict compliance with the guidelines set forth in the Guide for the Care and Use of Laboratory Animals issued by the National Institutes of Health (No. 2023AE1072). The *Zbp1* gene knockout mice (*Zbp1^−/−^*, Strain NO. T029037), *Zbp1 floxed* mice (*Zbp1^fl/fl^*, Strain NO. T019676), Lyz2‐Cre mice (Strain NO. T003822), and wild‐type (WT) mice with a C57BL/6J background were obtained from GemPharmatech Co., Ltd (Nanjing, China). To generate myeloid specific *Zbp1* gene knockout mice, *Zbp1^fl/fl^* mice were bred with Lyz2‐Cre mice. First, we crossbred Lyz2‐Cre mice with *Zbp1^fl/fl^* mice to generate heterozygote (*Zbp1^fl/+^* Lyz2‐Cre *^+/−^* mice). Next, male heterozygote were bred with female heterozygote to generate *Zbp1^fl/fl^* Lyz2‐Cre *^+/−^* mice (myeloid specific *Zbp1* gene knockout mice, abbreviated as *Zbp1^CKO^*) and *Zbp1^fl/fl^* (as the control counterpart of *Zbp1^CKO^*). Mice were maintained in a room featuring a stable temperature and a light‐dark cycle, and were fed a standard rodent diet. Random grouping was employed during the experimental setup. All animal experiments were conducted and analysed by a blinded researcher.

For animal model of sepsis‐induced myocardial dysfunction, *Zbp1^−/−^, Zbp1^CKO^, Zbp1^fl/fl^*, and 8–10‐week‐old WT male mice were administered an intraperitoneal injection of LPS (10 mg/kg, *Escherichia coli* O111:B4, #2630, Sigma‐Aldrich). Survival was monitored continuously for 7 days. After the experiment, all mice were euthanised under inhalation anaesthesia with isoflurane (1.5%–2%) and then heart tissues were rapidly extracted.

For pharmacological inhibition of STAT1, WT male mice received daily intraperitoneal injections of fludarabine (Flu, 50 mg/kg, HY‐B0069, MCE) for 5 days before LPS administration, while control group received an equal volume of solvent (Sol, 10% DMSO + 45% PEG300 + 5% Tween80 + 40% saline). Then, WT male mice were administered an intraperitoneal injection of LPS (10 mg/kg). Upon completion of the experiment, all mice were euthanised under inhalation anaesthesia with isoflurane (1.5%–2%) and then heart tissues were rapidly extracted.

### 2.2 Genotyping

DNA was extracted from mouse tail tissues using alkaline lysis method (50 mM NaOH), and the target DNA fragments were amplified by PCR. Primers used in PCR are listed in Table S1. The presence or absence of specific bands in gel electrophoresis indicates the genotype of the mice. Each genotype will typically produce a distinct banding pattern on the gel. The genotype of each mouse was then recorded for subsequent experiments.

### 2.3 Echocardiography

Transthoracic echocardiography was performed at predefined time points to evaluate cardiac function using the Vevo 2100 system (FUJIFILM VisualSonics, Inc., Toronto, Canada). M‐mode images of the left ventricle were captured and left ventricular diameter (LVID), anterior wall thickness (LVAW), and posterior wall thickness (LVPW) in both diastolic and systolic phases were measured. The ejection fraction (EF%) and fractional shortening (FS%) were determined using accompanying analysis software.

### 2.4 Cell culture

Murine HL‐1 cardiomyocytes (CM) were acquired from Chinese Academy of Sciences (Shanghai, China) and grown in Dulbecco's modified eagle medium (DMEM, Wisent, 319‐005‐CL) with 10% fetal bovine serum (FBS, Gibco, 10099141C) and 1% antibiotics (penicillin/streptomycin, Wisent, 450‐201‐EL) at 37°C in a humidified 5% CO_2_ atmosphere.

### 2.5 Mouse bone marrow‐derived macrophages isolation and culture

Bone marrow‐derived macrophages (BMDM) isolation was performed following established isolation protocols.^13^ The femurs and tibias of mice were flushed to collect bone marrow cells. Filter the flushed cells and remove red blood cell. Discarding the supernatant, the cells were resuspended and incubated with recombinant mouse macrophage colony‐stimulating factor (M‐CSF; Gibco, 315‐02). Cells were continuously exposed to M‐CSF at a final concentration of 10 ng/mL throughout the culture period to promote differentiation into mature macrophages, preparing them for subsequent experiments.

### 2.6 Transcriptome

Mice was administrated by LPS or saline for 24 h. RNA‐seq of heart tissues (three biological replicates per group) was conducted using the illumina Novaseq 6000 platform by LC‐Bio Technology CO., Ltd., (Hangzhou, China) in accordance with the manufacturer's guidelines. In the data analysis, a *P*‐adjusted value of 0.05 was used as the threshold to identify differentially expressed genes (https://bioconductor.org/packages/release/bioc/html /edgeR.html).

BMDM was administrated by LPS or phosphate buffer saline (PBS) for 24 h. Total RNA of BMDM (three biological replicates in each group) was prepared with trizol reagent (Invitrogen) and RNA‐seq was performed on NovaSeq X Plus by LC‐Bio Technology Co. Ltd (Shanghai, China). For data analysis, a *P*‐adjusted value of 0.05 was set as the threshold for identifying differentially expressed genes (http://geneontology.org/).

### 2.7 Single‐cell mRNA sequencing

A single‐cell mRNA sequencing (scRNA‐seq) was performed in hearts from LPS‐treated (LPS) and untreated WT (Ctrl) mice using cell suspension analysis (for each group, single‐cell suspensions from three hearts were pooled as one sample). Single cells were dissociated from tissues using a dissociation solution, containing 120 units/mL DNase I, 2 mg/mL papain, 0.35% collagenase IV, at 37°C with continuous shaking. The digestion process was halted by PBS with 10% FBS, followed by 5–10 cycles of pipetting with a Pasteur pipette. Filter the cell suspension, centrifuge it, and resuspended the pellet after removing red blood cells and dead cells. In order to confirm cell viability, trypan blue exclusion was performed, requiring a viability of more than 85%.

Single‐cell suspensions were applied to MobiNova‐100 Single‐Cell System to capture 10,000 individual cells, following the guidelines provided in the MobiCube Single‐Cell 3′RNA‐seq Kit manual. cDNA amplification and library conduction preparation were performed following the standard experimental procedures. The libraries sequenced on the Illumina NovaSeq 6000 platform (paired‐end multiplexing run) by LC‐Bio Technology Co. Ltd. (HangZhou, China), with a minimum sequencing depth of 20,000 reads/cell. The raw sequencing data were processed for demultiplexing and subsequently converted into FASTQ format using Illumina bcl2fastq software. MobiVision pipeline (https://www.mobidrop.com/en/bioinformatics/mobivision, version 3.2) was utilised for sample demultiplexing, barcode recognition, and quantification of single‐cell 3′ gene expression. The output from MobiVision was subsequently imported into Seurat (version 4.1.0) for further computational analyses, including dimensionality reduction, cell clustering, and comprehensive single‐cell RNA‐seq data exploration.

### 2.8 Single‐nucleus mRNA sequencing

A single‐nucleus mRNA sequencing (snRNA‐seq) was performed in hearts from LPS‐treated (LPS) or untreated WT (Ctrl) mice (for each group, single‐nucleus suspensions from three hearts were pooled as one sample) and LPS‐treated *Zbp1^−/−^* mice and WT littermates (for each group, single‐nucleus suspensions from three hearts were pooled as one sample) by using cell nuclei extraction analysis. The isolation of single nuclei was performed using Nuclei EZ Lysis buffer to prevent degradation. Reaction was stopped with ice‐cold 4% bovine serum albumin (BSA), followed by centrifugation at 300 g for 5 min at 4°C. Then, pellet was resuspended in lysis buffer with 4% BSA, de‐fragmented using Miltenyi Debris Removal Solution, and washed with buffer. Then centrifuge, filter the cell suspension, and resuspended. Single‐nuclei suspensions were applied to MobiNova‐100 Single‐Cell System to capture 10,000 individual nuclei, following the guidelines provided in the MobiCube Single‐Cell 3′RNA‐seq Kit manual. The next steps were the same as single‐cell sequencing.

### 2.9 Flow cytometry analysis

For cardiac flow cytometry analysis, mice were euthanised and their hearts were subsequently removed. The hearts were minced into small pieces and digested with Liberase DH Research Grade (Sigma, 05401054001) at 37°C for 30–40 min. Following the digestion of heart tissues, a single‐cell suspension was prepared using a 100 µm cell strainer. In the beginning, the cells were treated with Fc block (anti‐CD16/CD32, E‐AB‐F0997A, Elabscience) for 10 min at 4°C to prevent nonspecific antibody binding. Fluorochrome‐conjugated antibodies against F4/80 (E‐AB‐F0995D, Elabscience) and CD11b (E‐AB‐F1081C, Elabscience) were then added. For intracellular marker CD206 (E‐AB‐F1135H, Elabscience) staining, the cells were fixed with a fixation buffer and permeabilised with a permeabilisation buffer before incubation with the fluorochrome‐conjugated antibody against CD206. Finally, wash and resuspend in an appropriate volume for flow cytometry analysis by FACS Aria flow cytometer (BD Biosciences).

### 2.10 Lentivirus construction and infection

The knockdown of STAT1 in BMDM was accomplished using lentiviral‐based specific shRNA (sense sequence: 5′‐GATCCGCTGTTACTTTCCCAGATATTCTCGAGAATATCTGGGAAAGTAACAGCTTTTTTG‐3′, anti‐sense sequence: 5′‐AATTCAAAAAAGCTGTTACTTTCCCAGATATTCTCGAGAATATCTGGGAAAGTAACAGCG‐3′) constructed and purified by Hanbio Tech. For the purpose of mock transduction, an empty lentiviral vector (negative control, NC) lacking shRNA expression was used. BMDM was infected with the lentiviruses for 24 h.

### 2.11 Western blotting

Protein extraction from cells or tissues was performed using RIPA buffer (KeyGEN, KGB5303) along with protease inhibitors (KeyGEN, KGB5303). Samples of 20 µg were loaded onto sodium dodecyl sulfate (SDS)‐polyacrylamide gel electrophoresis gels and transferred to polyvinylidene difluoride membranes. Membranes were blocked and incubated with anti‐ZBP1 antibody (1:1000, AG‐20B‐0010‐C100, Adipogen), anti‐Tubulin antibody (1:2000, 11224‐1‐AP, Proteintech), anti‐GAPDH antibody (1:2000, FD0063‐50, Famacs) or anti‐STAT1 antibody (1:1000, 14994, CST). Following washing steps, membranes were subjected to a 60‐min incubation at ambient temperature with species‐matched HRP‐conjugated secondary antibodies (Horseradish peroxidase‐labelled goat anti‐rabbit IgG(H+L): 1:5000, RS0002, Immunoway; Horseradish peroxidase‐labelled goat anti‐mouse IgG(H+L): 1:5000, RS0001, Immunoway). The results were detected by enhanced chemiluminescence (Yeasen, 36208ES76) and quantified with ImageJ software.

### 2.12 Histological and immunohistochemistry analysis

Heart tissue sections with 5 µm thickness were obtained and histological evaluation was then carried out using haematoxylin and eosin (H&E) staining to assess myocardial architecture and inflammatory cell distribution.

To determine the distribution of inflammatory cells, we performed immunohistochemical staining for CD68, CD11b, F4/80, Ly6G, and Ly6C. After deparaffinisation and rehydration, the sections were heated in citrate buffer (pH = 6.0) for 2 min and then allowed to cool for 2 min, and this process was repeated three times for antigen retrieval. Upon reaching ambient temperature, the samples were treated with peroxidase inhibitor solution for 10 min to quench the endogenous enzyme activity. Subsequently, the sections were blocked and then incubated at 4°C with anti‐CD68 (1:200, ab283654, Abcam), anti‐CD11b (1:200, ab133357, Abcam), F4/80 (1:500, 70076, CST), Ly6G (1:2000, GB11229, Servicebio), and Ly6C (1:2000, ab314120, Abcam) antibodies. Following PBS washes, the sections were exposed to biotinylated goat anti‐mouse or anti‐rabbit IgG polymer for 20 min, followed by colour development using DAB substrate solution and counterstaining with hematoxylin for nuclear staining. Immunoreactive cells for CD68, CD11b, F4/80, Ly6G, and Ly6C were quantitatively evaluated for area% or number/view in three different areas of each heart tissue at 400× magnification under blinded conditions to assess the density of macrophages, neutrophils, and monocytes. All images were captured using a microscope (Leica, Germany).

### 2.13 Immunofluorescence

Frozen heart tissues were sectioned into continuous slices of 8 µm along the transverse plane. The sections were permeabilised, blocked and then incubated with anti‐CD68 (1:200, ab53444, Abcam), anti‐ZBP1 (1:200, NBP1‐76854, NOVUS), anti‐CD11b (1:200, ab133357, Abcam), anti‐α‐Actinin (1:200, A7811, Sigma Aldrich), anti‐inducible nitric oxide sythase (iNOS) (1:50, ab49999, Abcam) and anti‐CD206 (1:100, 24595, CST) antibodies. An equal amount of PBS was used as the negative control. After incubation, appropriate secondary antibodies were applied at room temperature, and cell nuclei were stained with 4′,6‐diamidino‐2‐phenylindole (DAPI) for 5 min. Using ImageJ software under blinded conditions, the number of double‐positive cells was counted in three high‐power fields per heart slice. The relative expression of ZBP1 in macrophages or CM was represented as the percentage of double‐positive cells per high‐power field in cardiac tissue, calculated by the formula: percentage of double‐positive cells = number of double‐positive cells per high‐power field/number of cell nuclei per high‐power field × 100%. As for the calculation of the proportion of macrophages with M1 and M2 phenotypes: percentage of M1/M2 macrophages = number of iNOS‐positive or CD206‐positive cells per high‐power field/number of cell nuclei per high‐power field × 100%. All images were captured using a confocal microscope (Olympus, Japan).

### 2.14 Genomic sequencing data analysis

The chromatin immunoprecipitation (CHIP)‐seq data of H3K27ac and STAT1 in BMDM were obtained from GEO database (GSE56121). The initial processing of the raw sequencing data was performed using the trim‐galore tool to remove low‐quality reads. Next, the high‐quality reads were mapped to the mm10 genome assembly with Bowtie2 and further deduplicated using Picard tools. Prior to visualisation, deduplicated reads were indexed using samtools and converted to bigwig format with the following settings: normalise using RPGC‐effective Genome Size 2652783500‐ignore Duplicates. The IGV software was used for data visualisation.

### 2.15 Dual luciferase reporter assay

The cells were cultured to a density of 90% in 24‐well plates. The pGL3 recombinants containing the *Zbp1* gene promoter (400 ng), pcDNA3.1‐STAT1 (400 ng), and pRL‐TK (100 ng) plasmids were transiently transfected into cells using the Lipofectamine 3000 reagent (Invitrogen, Carlsbad, CA, USA). Following a 48‐h incubation period, cell lysates for the reporter assay were prepared according to the protocol of the Dual‐Luciferase Reporter Assay System (Promega, Madison, WI, USA). Firefly luciferase activity (LUC) and Renilla luciferase activity (TK) were then quantified following the supplier's recommended procedures.

### 2.16 Statistical analysis

All statistical analyses were conducted with GraphPad Prism version 9.0. Data were presented as the mean ± standard error of mean (SEM). For survival analysis, Kaplan–Meier survival curves were generated and compared using the log‐rank test. Normality of the data distribution was verified through Kolmogorov–Smirnov testing and quantile–quantile (*Q*–*Q*) plot evaluation. Parametric tests were subsequently applied: two‐group comparisons employed Student's *t*‐test, while multi‐group analyses utilised one‐way ANOVA followed by Bonferroni post hoc correction. Statistical significance was defined as *P* < 0.05.

## 3 RESULTS

### 3.1 Z‐DNA binding protein 1 is upregulated by lipopolysaccharide in heart tissues of mice and is mainly distributed in macrophages

**FIGURE 1:**
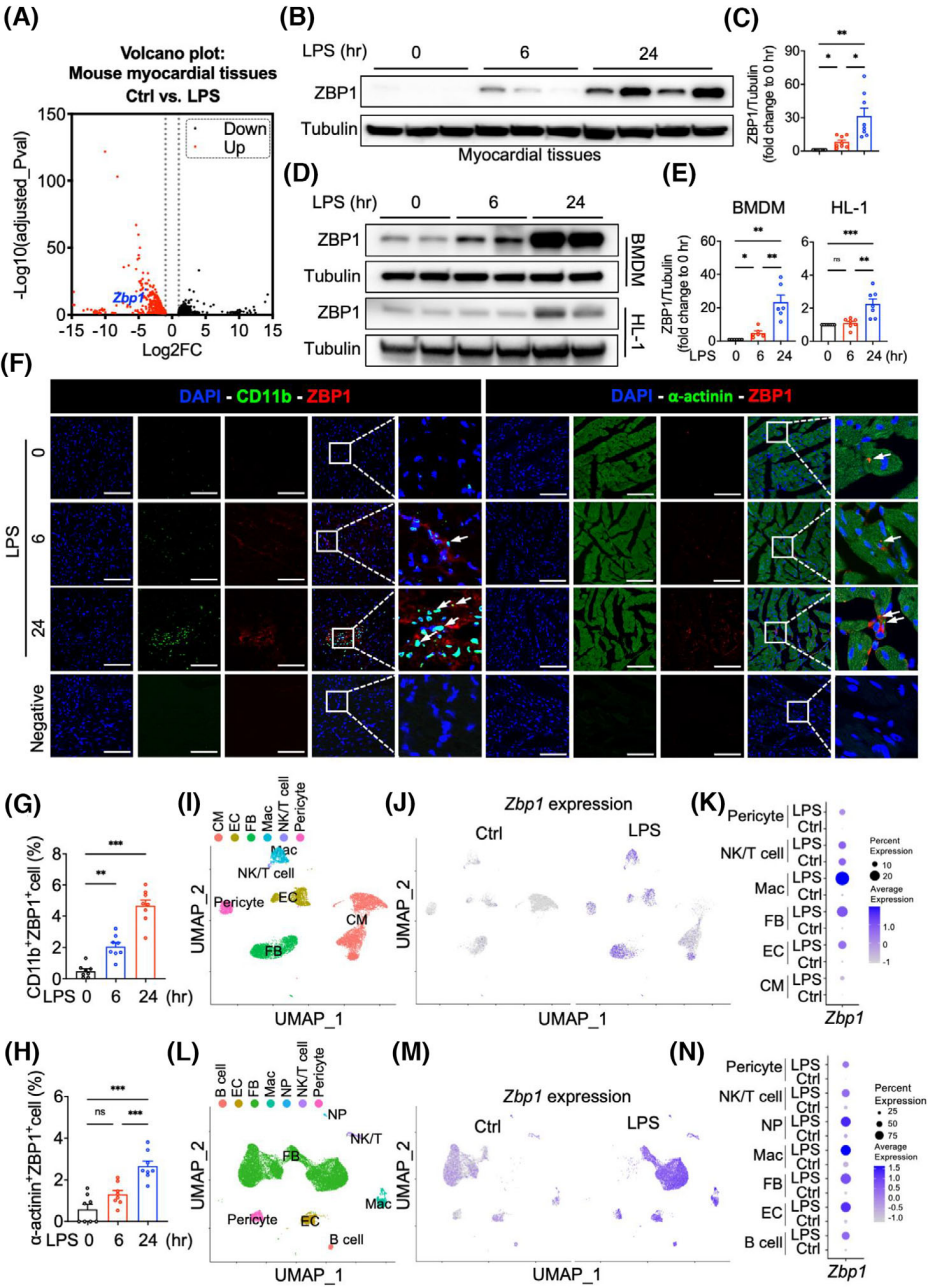
ZBP1 is upregulated in myocardial tissues of LPS‐induced mice and is mainly distributed in macrophages. (A) The volcano plot of RNA‐seq shows that differentially expressed genes between the myocardial tissues of LPS mice and WT mice. Up means upregulated gene in LPS vs. control‐treated hearts. (B,C) Western blotting detection and quantitative analysis of ZBP1 protein levels in myocardial tissues at course time post intraperitoneal injection of LPS (10 mg/kg; 0, 6, or 24 h) in WT mice (*n* = 8 in each group). The representative image shows data from a single batch of mice. (D,E) Western blotting detection and quantitative analysis of ZBP1 protein levels in BMDM or HL‐1 cells at course time post LPS administration (10 µg/mL; 0, 6, or 24 h; *n* = 6–7 in each group). (F) Representative images of the immunofluorescent staining of CD11b (left) or α‐actinin (right) and ZBP1 in hearts at course time post‐intraperitoneal injection of LPS in WT mice (10 mg/kg; 0, 6, or 24 h). White arrow indicates co‐localisation of ZBP1 in macrophages (left) or cardiomyocytes (right). [scale bar = 100 µm, *n* = 8 in each group]. (G,H) Quantitative analysis of ZBP1^+^ macrophages (G) or ZBP1^+^ cardiomyocytes (H) in hearts at course time post‐intraperitoneal injection of LPS in WT mice (*n* = 8 in each group). (I) UMAP plot of cell clusters in myocardial tissues from WT+ normal saline (NS, Ctrl) and WT+LPS (LPS) mice by using cell nuclei extraction analysis. (J,K) UMAP plot and dot plot of *Zbp1* expression in myocardial tissues from Ctrl and LPS mice. (L) UMAP plot of cell clusters in myocardial tissues from Ctrl and LPS mice by using cell suspension analysis. (M,N) Uniform manifold approximation and projection (UMAP) plot and dot plot of *Zbp1* expression in myocardial tissues from Ctrl and LPS mice. CM, cardiomyocyte; EC, endothelial cell; FB, fibroblast; Mac, macrophage; NP, neutrophil; WT, wild‐type; ZBP1, Z‐DNA binding protein 1; LPS, lipopolysaccharide; BMDM, bone marrow‐derived macrophages. Mean ± SEM; *P < 0.05, **P < 0.01, ***P < 0.001, ns, no significance.

After sepsis, a large number of macrophages were recruited into the heart, becoming the predominant immune cells in the heart.^14^ Recruited macrophages in the myocardium have the characteristics of interferon stimulation and pro‐inflammatory function.^15^ ZBP1 is known to be highly expressed in macrophages, which is relative with the pro‐inflammatory phenotype and interferon response.^8^, ^16^ However, there is a notable lack of studies demonstrating a link between ZBP1 and septic cardiomyopathy. Here, we found an increased expression of *Zbp1* in a transcriptome dataset of the myocardium of LPS‐induced mice (Figure 1A). Next, we validated that ZBP1 was time‐dependently upregulated in LPS‐induced myocardial tissues (Figure 1B,C). The protein levels of ZBP1 were also time‐dependently increased in LPS‐induced BMDM and HL‐1 cells (Figure 1D). The up‐regulation of ZBP1 was particularly more significant in BMDM (Figure 1D,E). Similar changing patterns were observed in immunofluorescent staining of heart sections. Increased immunoreactivity of ZBP1 was predominantly noted in CD11b^+^ or CD68^+^ macrophages rather than α‐actinin^+^ CM (Figures 1F–h and S1a,b). To gain a more comprehensive understanding of the changing profile in ZBP1 expression induced by LPS, we carried out snRNA‐seq and scRNA‐seq in hearts of mice to examine the distribution changes. In snRNA‐seq, 12,619 cells from two groups of hearts were analysed. Utilising specific marker genes (Figure S2a), we identified six cell clusters in hearts, including macrophage (Mac), fibroblast (FB), endothelial cell (EC), CM, NK/T cell, and pericyte (Figure 1I). Among these, the proportion of macrophage was increased prominently (Figure S2b,c). Notably, *Zbp1* mRNA in CM, EC, FB, and Mac appeared to be elevated by LPS (Figures 1J,K and S2d,e), which is partially consistent with immunofluorescence. In scRNA‐seq, 21,899 cells from two groups of hearts were analysed. Utilising specific marker genes (Figure S3a), we identified seven cell clusters in hearts, including B cell, EC, FB, Mac, Neutrophil (NP), NK/T cell, and pericyte (Figure 1l). The proportion of macrophage increased the most (Figure S3b,c). Similar with snRNA‐seq data, *Zbp1* mRNA in EC, FB, Mac, and pericyte appeared to be elevated by LPS; otherwise, scRNA‐seq results showed that *Zbp1* mRNA in B cell, NP, and NK/T cell was elevated by LPS (Figures 1M,N and S3d‐e). In snRNA‐seq analysis, we identified cardiac resident macrophages (Res_Mac) and infiltrating monocyte‐derived macrophages (MOMFs) using specific marker genes (Figure S4a,b). Following the LPS treatment, the proportion of MOMFs was significantly elevated (Figure S4c). Upon LPS stimulation, the *Zbp1* expression increased in both Res_Mac and MOMFs, with a more pronounced elevation observed in MOMFs (Figure S4d–f). Similar results were observed in scRNA‐seq analysis (Figure S5). Taken together, these results prove that ZBP1 is upregulated in myocardial tissues of LPS‐induced mice, which may be closely related to infiltrated macrophages.

### 3.2 Deletion of Z‐DNA binding protein 1 protects against septic myocardial dysfunction

**FIGURE 2:**
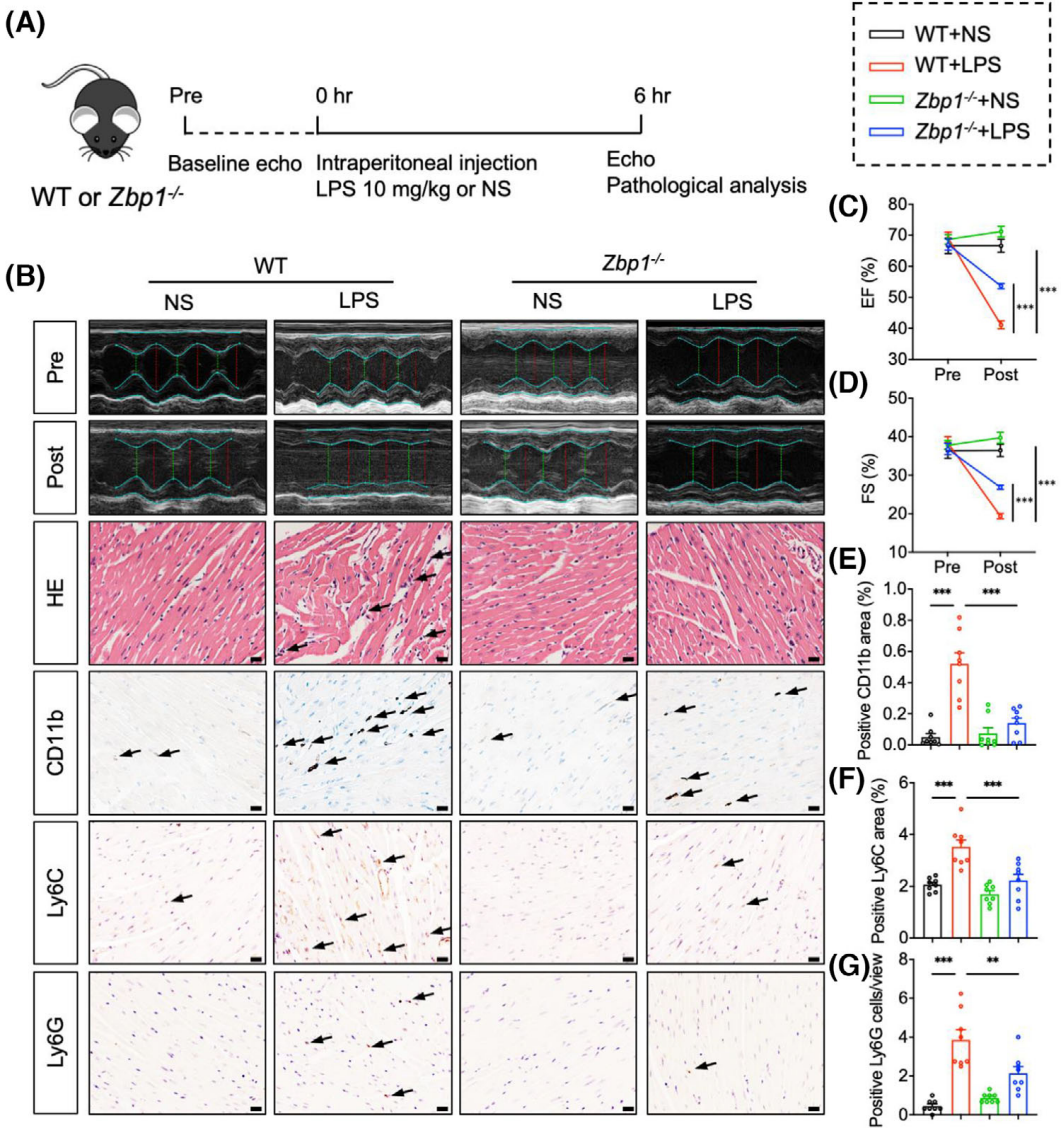
Deletion of ZBP1 protects against septic myocardial dysfunction. (A) Schematic showing the experimental design to investigate the impact of *Zbp1* deficiency on septic myocardial dysfunction (LPS: 10 mg/kg; 6 h). (B) Representative images of transthoracic M‐mode echocardiographic trance, hematoxylin and eosin (H&E) staining, immunohistochemistry staining of CD11b, Ly6C and Ly6G at 6 h after intraperitoneal injection of saline or LPS in WT or *Zbp1^−/−^* mice. Black arrow indicates positive area of cardiac injury, CD11b, Ly6C and Ly6G. [Scale bar = 20 µm, *n* = 8 in each group]. (C,D) M‐mode analysis of ejection fraction (EF) and fractional shortening (FS) before (pre) and 6 h after (post) LPS treatment (*n* = 8–9 in each group). (E–G) Quantitative analysis of CD11b^+^, Ly6C^+^ area and Ly6G^+^ cells in myocardial tissues (*n* = 8 in each group). Mean ± SEM; **P < 0.01, ***P < 0.001. ZBP1, Z‐DNA binding protein 1; LPS, lipopolysaccharide.

We further investigated the roles of ZBP1 in cardiac dysfunction, myocardial injury and inflammatory cell infiltration of LPS‐treated mice by using *Zbp1* knockout mice. We confirmed the susceptibility of cardiac function to sepsis in mice and detected a substantial reduction in left ventricular EF and FS, indicative of cardiac dysfunction at 6 h after LPS injection (Figure S6a–c). Next, the tail DNA of *Zbp1* knockout mice (*Zbp1^−/−^*) or littermate WT mice were detected to analyse the genotyping (Figure S7a,b). We also verified the knockout efficiency of ZBP1 in hearts of these mice (Figure S8a). As shown in Figure 2A, *Zbp1^−/−^* and littermate WT mice were subjected to LPS (10 mg/kg) for 6 h. Using echocardiography, we first examined the cardiac function. The results showed that *Zbp1* gene knockout significantly improved cardiac function of LPS‐treated mice (Figure 2B–D), as evidenced by EF and FS. In addition, we recorded the 7‐day survival rate of mice and found a significant decline in survival in WT + LPS group after LPS injection, while the survival rate in *Zbp1^−/−^* + LPS group was significantly improved (Figure S8b). As shown in Figure 2B, *Zbp1^−/−^* improved LPS‐induced disorder of cardiac muscle fibres in H&E staining. Next, heart sections were subjected to immunohistochemistry to investigate inflammatory cell infiltration (Figure 2B). As shown in Figure 2B,E–G, LPS‐stimulated WT mice showed obvious inflammatory cell infiltration, including CD11b, CD68, or F4/80 (Figure S8c–e)‐labelled macrophages, Ly6C‐labelled monocytes and Ly6G‐labelled neutrophils, while *Zbp1* gene knockout significantly inhibited the infiltration of above inflammatory cells. Overall, these results reveal that ZBP1 deficiency exerts cardioprotective effect on LPS‐induced cardiac dysfunction, myocardial injury and inflammatory cell infiltration.

### 3.3 Z‐DNA binding protein 1 alters the ratio of M1 and M2 macrophages in septic cardiomyopathy

**FIGURE 3:**
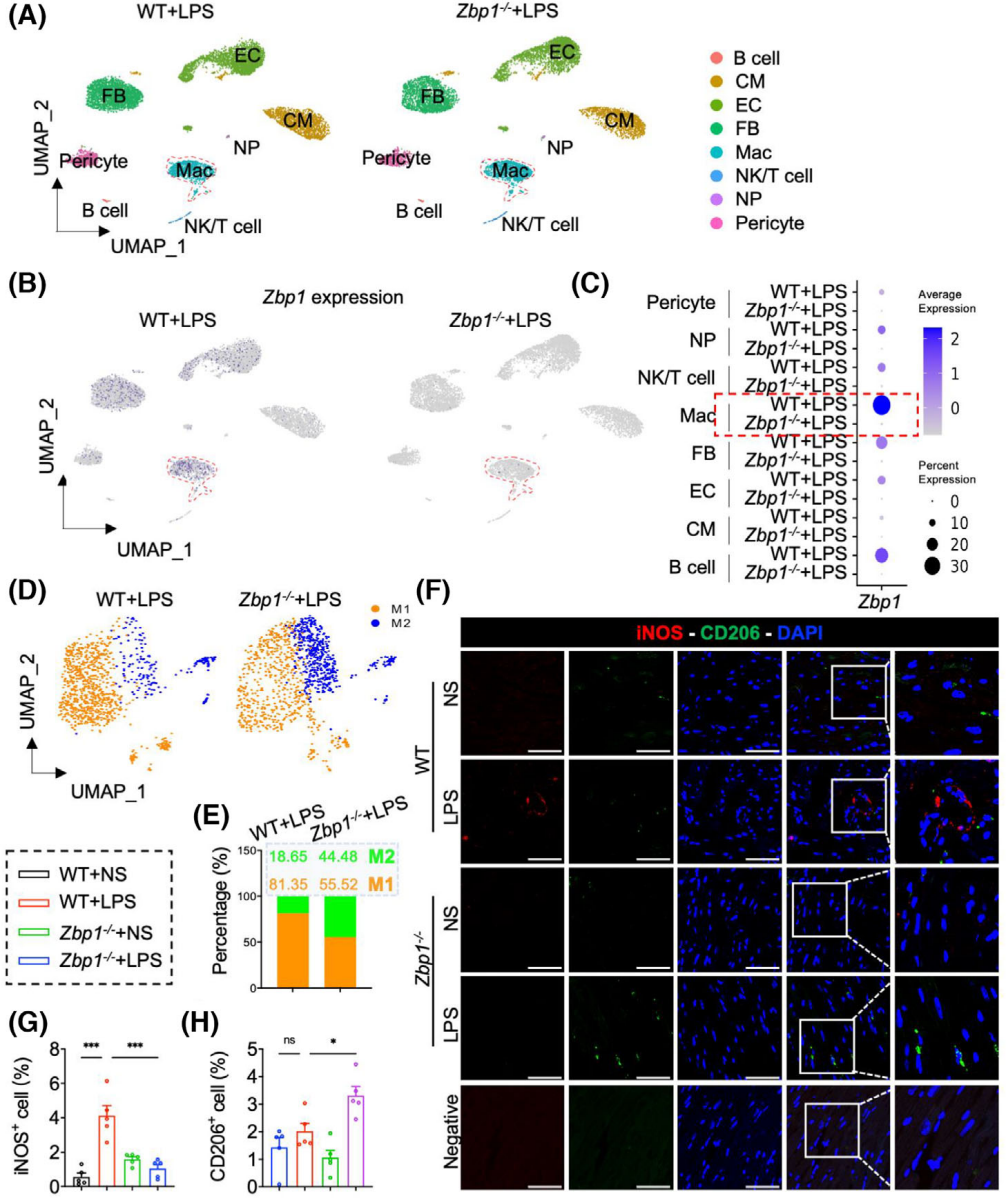
ZBP1 is mainly expressed in macrophages and alters the ratio of M1 and M2 macrophages in sepsis‐induced myocardial dysfunction. A single‐nucleus mRNA sequencing (snRNA‐seq) was performed in hearts from LPS‐treated WT or *Zbp1^−/−^* mice (for each group, single‐cell suspensions from three hearts were pooled as one sample). (A) UMAP plot of cell clusters in myocardial tissues from WT and *Zbp1^−/−^* mice induced by LPS. CM, cardiomyocyte; EC, endothelial cell; FB, fibroblast; Mac, macrophage; NP, neutrophil. (B,C) UMAP plot and dot plot of *Zbp1* expression in myocardial tissues from WT and *Zbp1^−/−^* mice induced by LPS. (D,E) UMAP plot of macrophage in myocardial tissues from WT and *Zbp1^−/−^* mice induced by LPS. M1, M1 macrophage; M2, M2 macrophage. (F–H) Heart tissues were labelled with M1 macrophage marker iNOS and M2 macrophage marker CD206. Tissues were counterstained with DAPI. Representative staining images (F) and quantification of positive cells (G,H) are shown [scale bar = 50 µm, *n* = 5]. Mean ± SEM; *P < 0.05, ***P < 0.001, ns, no significance. WT, wild‐type; ZBP1, Z‐DNA binding protein 1; LPS, lipopolysaccharide; snRNA‐seq, single‐nucleus mRNA sequencing.

As has been noted, Mac was the cell population with the largest increase in proportion following LPS treatment (Figures S2b,c and S3b,c). Moreover, ZBP1 expression was the most abundant in Mac among all cell populations (Figures S2d and S3d), and the elevation of *Zbp1* mRNA in Mac after LPS treatment was the most obvious as well (Figures 1J,K,M,N, and S2e and S3e). Next, in order to examine the changing profile of macrophages in septic myocardial dysfunction, we carried out a snRNA‐seq in hearts from LPS‐treated WT or *Zbp1^−/−^* mice. In total, 17,949 cells from two groups of hearts were analysed. Utilising specific marker genes (Figure S9a), we identified eight cell clusters in hearts, including B cell, CM, EC, FB, Mac, NP, NK/T cell and pericyte (Figure 3A). Notably, *Zbp1* mRNA in macrophages appeared to be the most abundant population within the dataset (Figure 3B,C), which is consistent with snRNA‐seq/scRNA‐seq data in Figure 1. Meanwhile, UMAP plot and Dotplot showed that *Zbp1* gene was deleted in *Zbp1^−/−^* hearts (Figure 3b,c).

Furthermore, using specific molecular markers (Figure S9b), we partitioned the macrophages into two main functional populations, including M1 macrophages and M2 macrophages (Figure 3D). It is worth noting that *Zbp1* gene knockout reduced the proportion of M1 macrophages (from 81.35% to 55.52%) and increased the proportion of M2 macrophages (from 18.65% to 44.48%) (Figure 3E). Double fluorescence staining of iNOS (M1 marker) and CD206 (M2 marker) also showed that deficiency of ZBP1 decreased the content of M1 macrophages but increased the M2 macrophages, which was consistent with the snRNA‐seq results (Figure 3F–H). Moreover, flow cytometric analysis of the heart further validated an increase in CD206^+^ CD11b^+^ F4/80^+^ cells (M2 macrophages) in the hearts of *Zbp1^−/−^* +LPS mice, compared with that in WT + LPS mice (Figure S9c). These data suggest that ZBP1 is mainly expressed in macrophages and alters the ratio of M1 and M2 macrophages in sepsis‐induced myocardial dysfunction.

### 3.4 Deletion of Z‐DNA binding protein 1 promotes the macrophage polarisation from M1 to M2 in sepsis‐induced myocardial dysfunction

**FIGURE 4:**
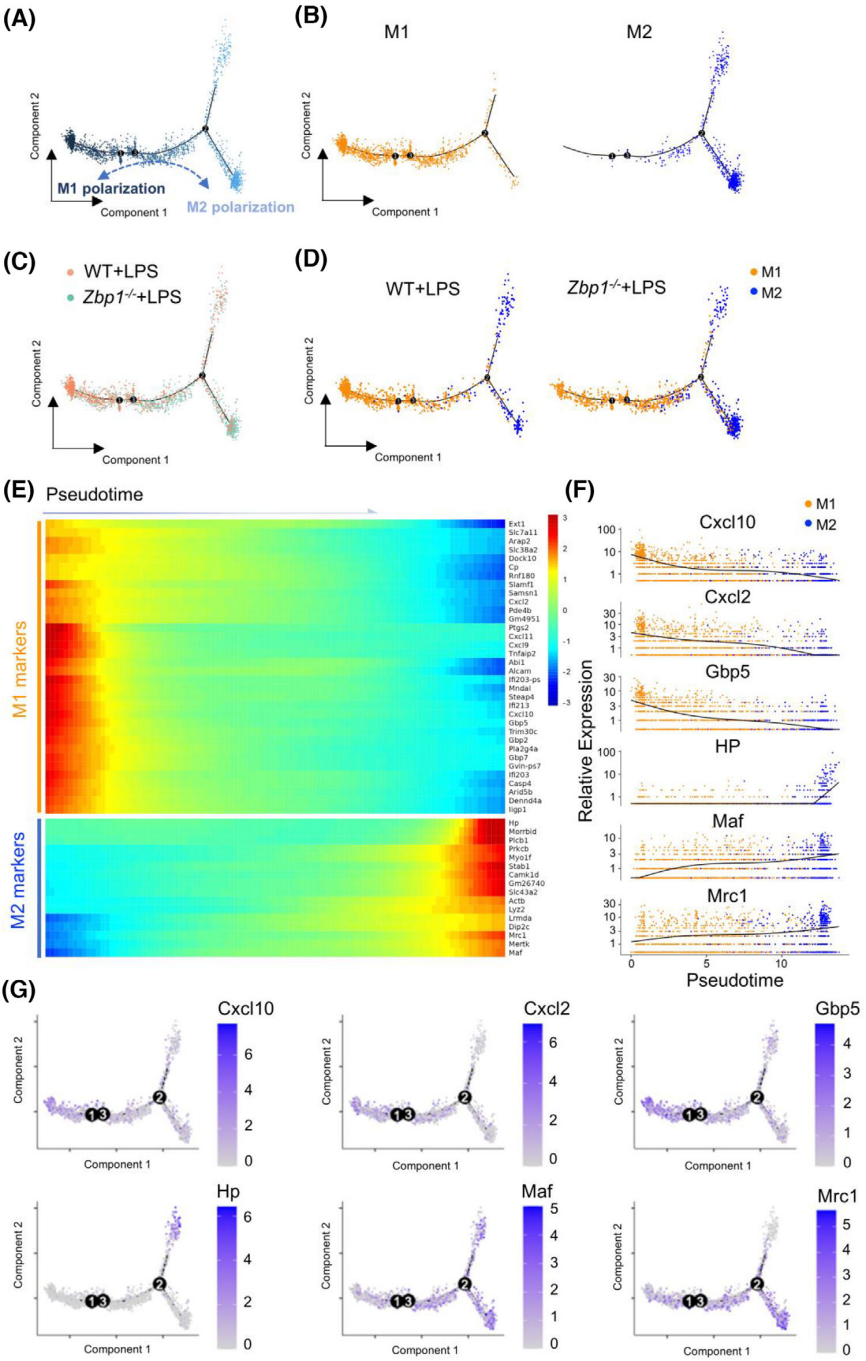
Pseudotime analysis reveals that deletion of ZBP1 promotes the macrophage polarisation from M1 to M2 in septic myocardial dysfunction. (A,B) The Monocle prediction of monocyte–macrophage compartment developmental trajectory with pseudotime mapped along. (C,D) The Monocle prediction of monocyte–macrophage compartment developmental trajectory split by WT+LPS and *Zbp1^−/−^* +LPS groups. (E) Heatmap reveals pseudotemporal expression pattern of clustering genes among macrophage populations in LPS mice. Pseudotime‐dependent marker genes following the similar kinetic trends are categorised into the same cluster. Expression scale is shown on the right. (F) Representative M1/M2 marker genes expression plotted along the pseudotime, with Seurat's cluster mapped along. (G) Representative M1/M2 marker genes expression of monocyte–macrophage compartment developmental trajectory with pseudotime mapped. WT, wild‐type; ZBP1, Z‐DNA binding protein 1; LPS, lipopolysaccharide.

During sepsis, LPS could induce macrophages to adopt a pro‐inflammatory phenotype (M1 macrophages) within the hearts.^4^ To investigate ZBP1‐dependent regulation on macrophage polarisation in septic myocardial dysfunction, we further constructed a single cell‐based differentiation trajectory from snRNA‐seq (Figure 4A). M1 or M2 macrophages are broadly distributed on the left or right sides of the trajectory branch, respectively (Figure 4B). As shown in Figure 4C, in the hearts of LPS mice, WT macrophages mainly aggregated in the end of the left‐side trajectory, while *Zbp1^−/−^* macrophages were primarily distributed at the right‐half of the major trajectory branch. It is significant to note that *Zbp1* gene knockout reduced the M1 macrophages distributed at the left of trajectory branch, while increasing the M2 macrophages aggregated in the right‐side trajectory (Figure 4D). These data suggest that deletion of ZBP1 promotes the macrophage polarisation from M1 to M2.

Next, we analysed the differentially expressed genes that co‐vary along pseudotime by Monocle2 to further investigate the genetic characteristic of macrophage polarisation in pseudotime trajectory. As shown in Figure 4E, M1 macrophage‐related genes were upregulated at the beginning of the cell trajectory, where WT macrophages were broadly located, including *Cxcl10*, *Cxcl2*, *Gbp5*, etc. On the contrary, highly expressed signatures in M2 macrophages, such as *HP*, *Maf*, *Mrc1*, etc., were greatly increased at the end stage of pseudotime trajectory, where *Zbp1^−/−^* macrophages mainly distributed (Figure 4E). In addition, to examine the direction of macrophage polarisation alongside the reconstructed trajectory from the left M1 to the right M2, we tracked the representative M1 and M2 marker genes expression changes during pseudotime (Figure 4F,G). The expression of M1 marker genes (*Cxcl10*, *Cxcl2* and *Gbp5*) were reduced alongside the pseudotime from M1 to M2, while the expression of the M2 marker genes (*HP*, *Maf* and *Mrc1*) were elevated in this process (Figure 4F,G). These results reveal that *Zbp1* gene knockout could inhibit the M1‐related genes transcription but increase the M2‐related genes expression.

### 3.5 Myeloid‐specific *Zbp1* deficiency protects against sepsis‐induced myocardial dysfunction

**FIGURE 5:**
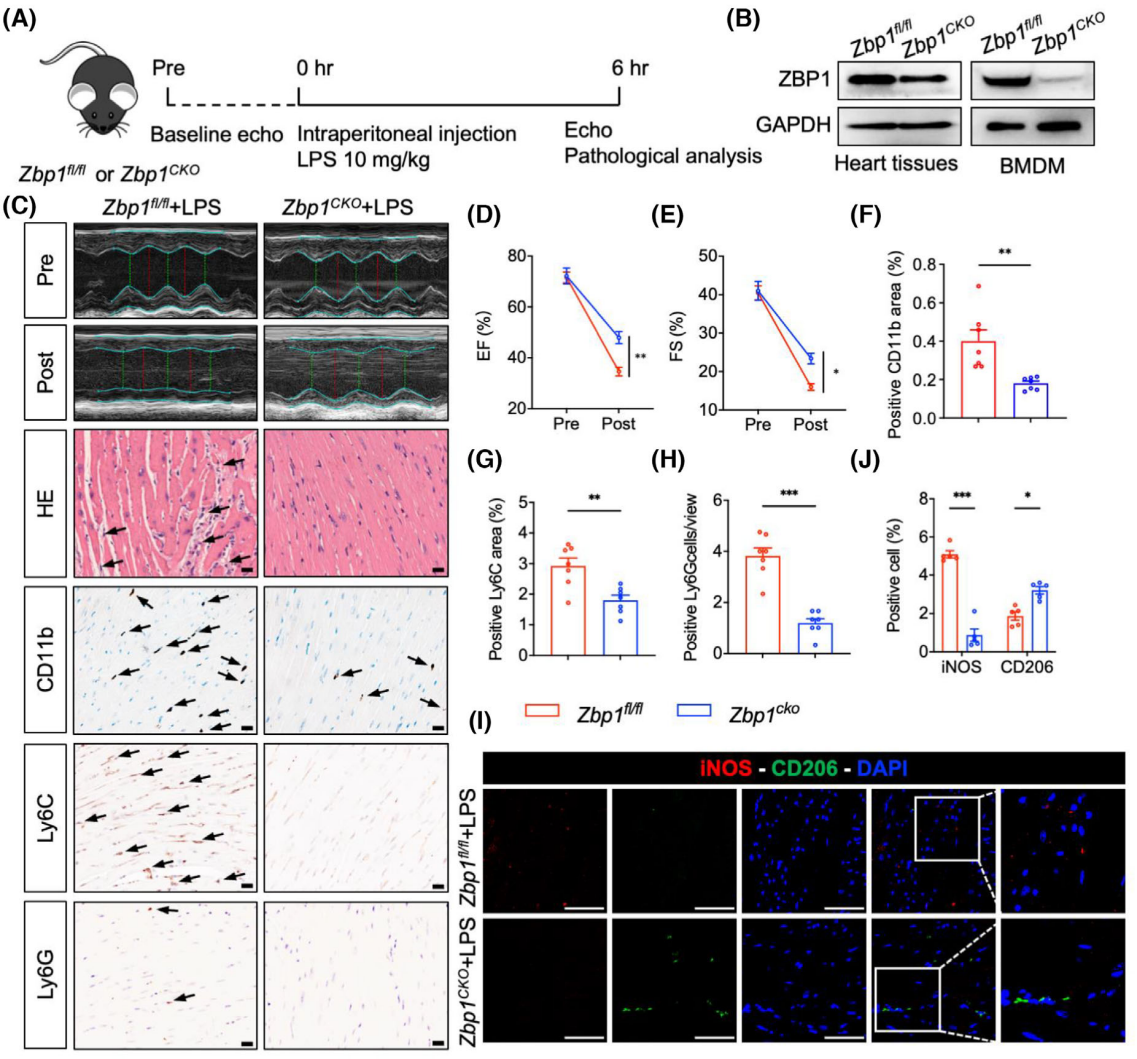
Myeloid‐specific *Zbp1* deficiency protects against sepsis‐induced myocardial dysfunction. (A) Schematic showing experimental design to investigate the impact of myeloid‐specific *Zbp1* deficiency on septic myocardial dysfunction (LPS: 10 mg/kg; 6 h). (B) *Zbp1* gene knockout efficiency in hearts and bone marrow cells was detected by Western blot. (C–E) Representative images of transthoracic M‐mode echocardiographic trance, hematoxylin and eosin (H&E) staining, immunohistochemistry staining of CD11b, Ly6C and Ly6G at 6 h after intraperitoneal injection of LPS in *Zbp1^fl/fl^* or *Zbp1^cko^* mice. M‐mode analysis of EF (D) and FS (E) before (pre) and 6 h after (post) LPS treatment. [scale bar = 20 µm, *n* = 7 in each group]. (F–H) Quantitative analysis of CD11b^+^, Ly6C^+^ area and Ly6G^+^ cells in myocardial tissues (*n* = 7 in each group). (I,J) Heart tissues were labelled with M1 macrophage marker iNOS and M2 macrophage marker CD206. Tissues were counterstained with DAPI. Representative staining images and quantification of positive cells are shown [scale bar = 50 µm, *n* = 5 in each group]. Mean ± SEM; *P < 0.05, **P < 0.01, ***P < 0.001. LPS, lipopolysaccharide; EF, ejection fraction; FS, fractional shortening; DAPI, 4′,6‐diamidino‐2‐phenylindole.

snRNA‐seq data indicated that macrophage‐derived ZBP1 could potentially be crucial in myocardial dysfunction induced by sepsis. To further investigate the role of macrophage‐derived ZBP1 in septic myocardial dysfunction, we generated a myeloid‐specific *Zbp1* knockout mice (*Zbp1^CKO^*) by crossing *Zbp1^fl/fl^* mice and Lyz2‐Cre mice. The tail DNA of *Zbp1^CKO^* mice or littermate *Zbp1^fl/fl^* mice were detected to analyse the genotyping (Figure S10a,b). *Zbp1^CKO^* mice and littermate *Zbp1^fl/fl^* mice (as the control counterpart of *Zbp1^CKO^*) were subjected to LPS administration (Figure 5A). We also verified the knockout efficiency of ZBP1 in BMDM of these mice (Figure 5B). Utilising echocardiography, we first examined the cardiac function, and the results showed that myeloid‐specific *Zbp1* knockout significantly improved cardiac function of LPS‐treated mice (Figure 5C–E), as evidenced by EF and FS. H&E staining in cardiac sections showed that *Zbp1^CKO^* abrogated LPS‐induced disorder of cardiac muscle fibres (Figure 5C). Next, heart sections were subjected to immunohistochemistry to investigate inflammatory cell infiltration. As shown in Figure 5C, F–H, *Zbp1^CKO^* significantly inhibited the infiltration of inflammatory cells, including CD11b, CD68, or F4/80 (Figure S10c–e)‐labelled macrophages, Ly6C‐labelled monocytes and Ly6G‐labelled neutrophils. Likewise, double fluorescence staining of iNOS (M1 marker) and CD206 (M2 marker) confirmed that *Zbp1^CKO^* decreased the content of M1 macrophages but increased the M2 macrophages (Figure 5I,J). Collectively, these observations validate that macrophage‐derived ZBP1 plays a detrimental role in septic myocardial dysfunction.

### 3.6 Lipopolysaccharide increases the transcription and expression of Z‐DNA binding protein 1 through signal transducer and activator of transcription 1 in macrophages

**FIGURE 6:**
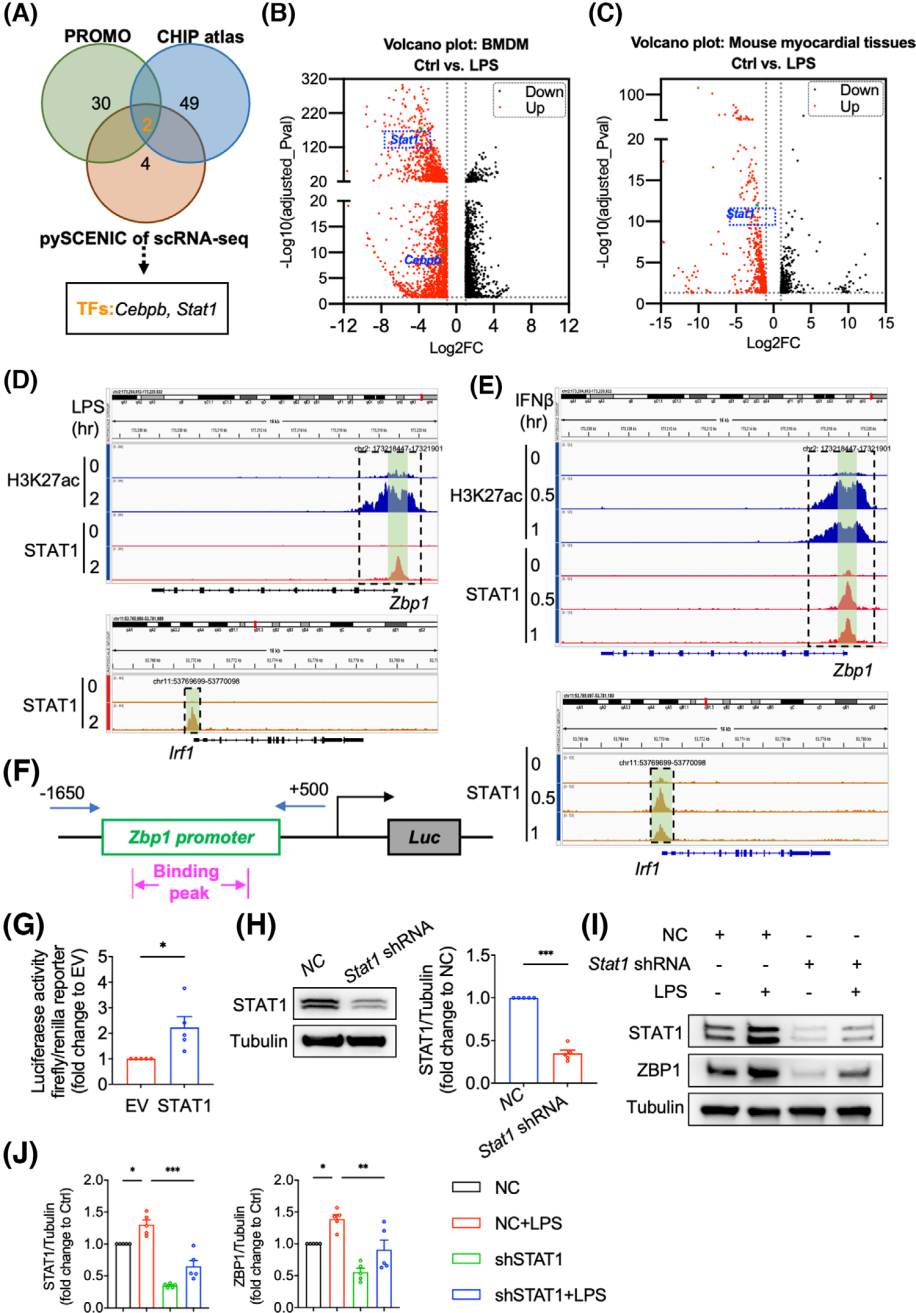
STAT1 promotes the transcription of ZBP1 in LPS‐induced macrophages. (A) PROMO database, CHIP atlas database and pySCENIC of snRNA‐seq were applied to predict the transcription factors (TFs) regulating ZBP1 expression. (B) The volcano plot of RNA‐seq in BMDM shows that LPS upregulates *Stat1* and *Cebpb* gene expression. (C) The volcano plot of RNA‐seq in mouse myocardial tissue shows that LPS upregulates *Stat1* gene expression. (D) The visualisation of enrichment of H3K27ac and STAT1 at *Zbp1* (upper) or *Irf1* (below) loci in LPS‐stimulated BMDM. Both of data were normalised using reads per genomic content (RPGC). The peak at transcript start site of *Zbp1* and *Irf1* gene were enclosed with dashed line. (E) The visualisation of enrichment of H3K27ac and STAT1 at *Zbp1* (upper) or *Irf1* (below) loci in IFNβ‐stimulated BMDM. (F,G) HEK293T cells were co‐transfected with the luciferases and STAT1 luciferase reporter plasmid for 48 h. Dual luciferase reporter assay detected the luciferase activation driven by *Zbp1* promoter after normalisation to Renilla luciferase (*n* = 5 independent experiments). (h–j) STAT1‐knockdown BMDMs achieved by short hairpin RNA (shSTAT1) were stimulated by LPS. Western blot analyses the protein level of ZBP1 (*n* = 5 independent experiments). Mean ± SEM; *P < 0.05, **P < 0.01, ***P < 0.001. ZBP1, Z‐DNA binding protein 1; LPS, lipopolysaccharide; BMDM, bone marrow‐derived macrophages; STAT1, signal transducer and activator of transcription 1; snRNA‐seq, single‐nucleus mRNA sequencing.

Finally, we explored the mechanism underlying the increased expression of ZBP1 in septic myocardial dysfunction. In general, protein content is mainly affected by gene expression and/or protein post‐translational modification. Here, we investigated the transcriptional activation of ZBP1 by PROMO database (Table S2), CHIP atlas database (Table S3) and pySCENIC of snRNA‐seq (Table S4). Through a comparison of PROMO, CHIP atlas and pySCENIC of snRNA‐seq, we identified two potential transcription factors of ZBP1, including STAT1 and Cebpb (Figure 6A). Among these two transcription factors, only STAT1 is both significantly increased in LPS‐induced BMDM (Figure 6B) and mouse myocardial tissues (Figure 6C). In addition, STAT1 acts as a central transcription mechanism underlying LPS‐mediated macrophage polarisation.^17^ Thus, we speculated that STAT1 might be the transcription factor to upregulate ZBP1 expression.

Next, we analysed a CHIP‐seq data of STAT1 and H3K27ac in BMDM from GEO database (GSE56121). As shown in Figure 6D, STAT1 bound to the promoter regions of *Zbp1* and *Irf1* (used as the positive control binding gene of STAT1) genes after LPS stimulation. Consistently, the promoter regions of *Zbp1* gene were associated with prominent acetylated H3K27 modification, indicating that STAT1 promotes the transcriptional activation of ZBP1. Similarly, the promoter regions of *Zbp1* gene could bind with STAT1 and be modified by H3K27ac under IFNβ stimulation (Figure 6E), indicating that STAT1 might be the critical transcription factor of ZBP1 in LPS‐stimulated BMDM. Next, we cloned the promoter regions of *Zbp1* and performed the luciferase promoter assay. As shown in Figure 6F,G, STAT1 increased *Zbp1* promotor‐drived luciferase activity. LPS significantly increased *Zbp1* gene expression in BMDM, while STAT1 knockdown (Figure 6H) abrogated the *Zbp1* up‐regulation induced by LPS (Figure 6I,J), validating that LPS increased the expression of *Zbp1* gene via STAT1. Collectively, these results reveal that STAT1 binds to the *Zbp1* promoter regions and activated *Zbp1* transcription in macrophages.

### 3.7 Blocking signal transducer and activator of transcription 1 prevents cardiac dysfunction caused by sepsis

**FIGURE 7:**
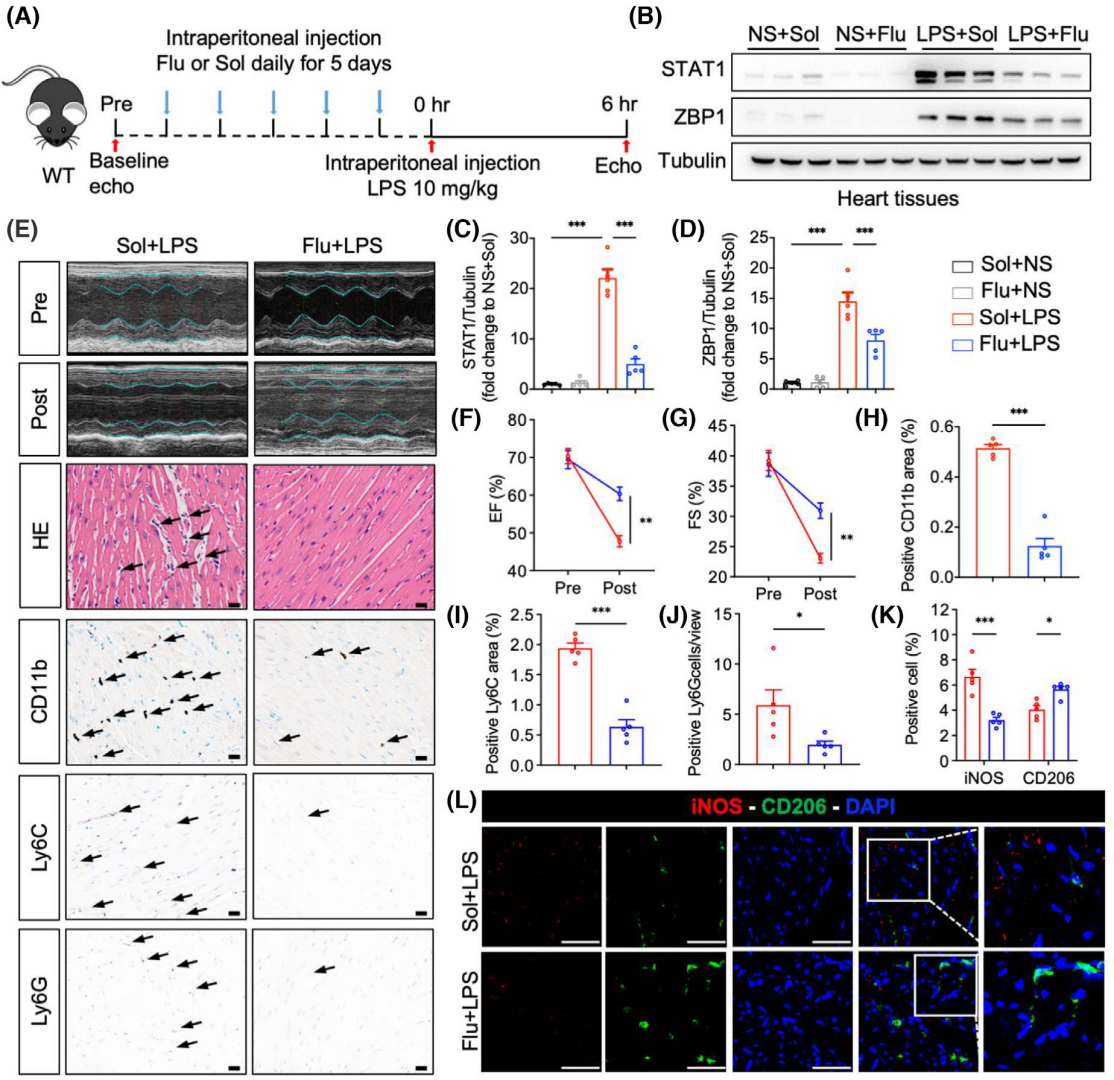
Blocking STAT1 prevents cardiac dysfunction caused by sepsis. (A) Schematic showing experimental design to investigate the impact of STAT1 inhibition on septic myocardial dysfunction (Flu, fludarabine, STAT1 inhibitor, 50 mg/kg/day; Sol, solvent). (B–D) Western blotting detection and quantitative analysis of STAT1 and ZBP1 in myocardial tissues at 6 h after intraperitoneal injection of NS or LPS (10 mg/kg) in solvent or fludarabine‐treated mice (*n* = 5 in each group). (E–G) Representative images of transthoracic M‐mode echocardiographic trance, hematoxylin and eosin (H&E) staining, immunohistochemistry staining of CD11b, Ly6C and Ly6G at 6 h after intraperitoneal injection of LPS in solvent or fludarabine‐treated mice. M‐mode analysis of EF (F) and FS (G) before (pre) and 6 h after (post) LPS treatment. [Scale bar = 20 µm, *n* = 8–9 in each group]. (H–J) Quantitative analysis of CD11b^+^, Ly6C^+^ area and Ly6G^+^ cells in myocardial tissues (*n* = 5 in each group). (k–l) Heart tissues were labelled with M1 macrophage marker iNOS and M2 macrophage marker CD206. Tissues were counterstained with DAPI. Representative staining images and quantification of positive cells are shown [scale bar = 50 µm, *n* = 5 in each group]. Mean ± SEM; *P < 0.05, ***P < 0.001. ZBP1, Z‐DNA binding protein 1; LPS, lipopolysaccharide; STAT1, signal transducer and activator of transcription 1; EF, ejection fraction; FS, fractional shortening; DAPI, 4′,6‐diamidino‐2‐phenylindole.

We additionally examined the effect of pharmacologically inhibiting STAT1 on cardiac dysfunction induced by sepsis. Fludarabine, a specific STAT1 inhibitor, was administered daily via intraperitoneal injection to mice for 5 consecutive days, after which these mice were challenged with LPS (Figure 7A). Initially, we confirmed the efficacy of STAT1 inhibition by fludarabine in LPS‐stimulated BMDM (Figure S11A,B) and myocardial tissues from LPS‐challenged mice (Figure 7B–d), resulting in a reduction of ZBP1 expression, consistent with findings observed in BMDM. Subsequently, we assessed cardiac function; the results indicated that compared to the Sol+LPS group, fludarabine treatment significantly improved cardiac function in LPS‐challenged mice, as evidenced by enhanced EF and FS (Figure 7E–G). H&E staining of cardiac sections indicated that fludarabine treatment effectively reversed the LPS‐induced disruption of cardiac muscle fibres (Figure 7E). Immunohistochemistry was performed on heart sections to assess inflammatory cell infiltration. As demonstrated in Figure 7E, H–J, fludarabine significantly reduced the infiltration of inflammatory cells, including CD11b, CD68, or F4/80 (Figure S11c–e)‐labelled macrophages, Ly6C‐labelled monocytes and Ly6G‐labelled neutrophils. Furthermore, double fluorescence staining of iNOS (M1 marker) and CD206 (M2 marker) confirmed that fludarabine treatment decreased the content of M1 macrophages but increased the M2 macrophages (Figure 7K,L). These observations validate that LPS upregulates ZBP1 expression through STAT1 in vivo and demonstrate that the STAT1 inhibitor effectively mitigates sepsis‐induced cardiac dysfunction.

## 4 DISCUSSION

**FIGURE 8:**
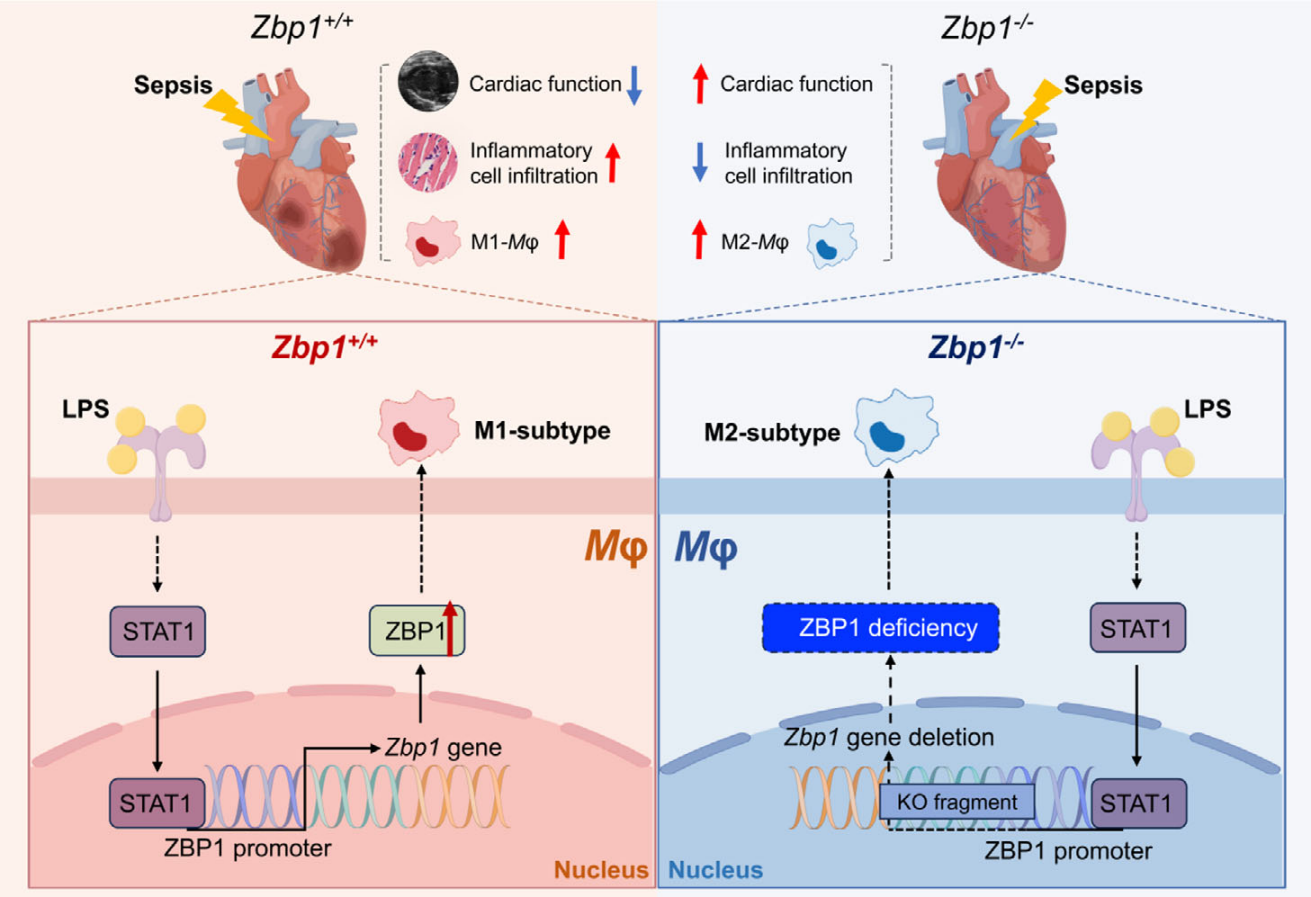
Loss of ZBP1 attenuates septic cardiomyopathy. LPS induces the transcription and expression of ZBP1 via STAT1, driving macrophage polarisation towards the pro‐inflammatory M1‐subtype macrophage, which results in inflammatory cell infiltration and cardiac dysfunction. In contrast, myeloid‐specific ZBP1 deficiency shifts macrophages towards the anti‐inflammatory M2 phenotype, reducing inflammatory cell infiltration and alleviating cardiac dysfunction. These findings highlight the role of macrophage‐derived ZBP1 in septic cardiomyopathy, suggesting that ZBP1 could be a potential therapeutic target for septic cardiomyopathy. *Mφ*, macrophage. KO fragment, knockout fragment of *Zbp1* gene. ZBP1, Z‐DNA binding protein 1; LPS, lipopolysaccharide; STAT1, signal transducer and activator of transcription 1.

During sepsis, a large number of macrophages infiltrate the myocardium and cause the initial cardiac inflammation.^4^ These pro‐inflammatory phenotype macrophages (M1 macrophages) prolong the inflammatory response, thus disrupting energy metabolism, stimulating excessive production of nitric oxide, unbalancing calcium homeostasis, and leading to myocardial dysfunction.^5^ Here, we present evidences that ZBP1 plays a dominant role in LPS‐induced M1 macrophage polarisation and sepsis‐induced myocardial dysfunction. We determine that in LPS‐induced myocardial tissues, ZBP1 is upregulated and is mainly expressed in macrophages. Loss of ZBP1 in macrophages protects heart against sepsis‐induced myocardial dysfunction and inflammatory cell infiltration. Mechanistically, we showed that ZBP1 promotes M1 macrophage polarisation and LPS increased the transcription of ZBP1 via STAT1. Our research emphasises the possibility of macrophage‐derived ZBP1 serving as a therapeutic target for septic cardiomyopathy and reveals a novel mechanism by which ZBP1 regulates macrophage activation (Figure 8).

ZBP1 was originally identified as a nucleic acid sensing protein via its Zα domain^6^ and was newly identified as a driver of PANoptosome assembly through its RHIM domain to instigate PANoptosis.^7^ In addition, ZBP1 was known to positively regulate macrophage inflammation by promoting LPS‐induced inflammasome activation and IL‐1β release in macrophages.^9^ Wang and colleagues found that ZBP1 was mainly upregulated in M1 macrophages compared to both M0 and M2 polarised macrophages.^10^ Further, gain‐ and loss‐function analysis revealed that ZBP1 could modulate the macrophage polarisation from M1 to M2.^10^ *Zbp1* deficiency significantly enhanced the survival rate of mice in the cecal ligation and puncture‐induced sepsis model.^18^ At present, there are limited reports on the direct regulation of ZBP1 in cardiac disorders. Lei et al. discovered that deletion of ZBP1 could protect hearts against doxorubicin‐induced cardiotoxicity and IFN‐I‐modulated cardiac inflammation.^11^ Besides, doxorubicin treatment could induce ZBP1 expression and IFN‐I responses in cardiac myeloid cells in vivo.^11^ In line with their findings, our work demonstrated that ZBP1 deficiency in macrophages exerted cardioprotective effect on LPS‐induced cardiac dysfunction, myocardial injury and inflammatory cell infiltration. Nevertheless, another research found that ZBP1 unexpectedly provided protection against the RIPK3‐NF‐κB‐NLRP3 pathway activation and inflammatory cytokine release induced by mitochondrial DNA in CM.^12^ This suggests that, even in the heart, different cellular sources of ZBP1 regulate the inflammatory response in opposite ways.

It is worth noting that ZBP1 depletion, both in whole‐body and macrophage‐specific contexts, partially rescues LPS‐induced cardiac dysfunction, which suggests that the pathogenesis of septic myocardial dysfunction is diverse and complex. Current academic perspectives indicate that multiple mechanisms are involved in septic cardiomyopathy, including inflammatory cell infiltration, CM death, etc.^3^ Even regarding the function of macrophages, various aspects such as migration, polarisation, and metabolism play significant roles.^4^ In addition, phenol‐purified LPS carries a risk of contamination with PAMPs, including bacterial genomic DNA. Consequently, it is challenging to distinguish between the role of ZBP1 in LPS‐induced sepsis and its recognition of contaminating DNA. While the primary objective of treating mice is to induce sepsis, the specific agent responsible for this induction—whether LPS or other contaminants—is not very critical to the model's main goal.

In terms of activation, M1 macrophages represent the classical activation mode, generally induced by pro‐inflammatory stimuli, including LPS or IFN‐γ.^19^ Although inflammatory cells account for only about 10% of the total heart,^20^ tissue resident and recruited circulating macrophages can regulate cardiac inflammatory response through macrophage polarisation, thereby affecting cardiac dysfunction and myocardial damage during sepsis.^21^ ZBP1 is mainly upregulated in M1 macrophages compared to both M0 and M2 polarised macrophages and modulates the M1 macrophage polarisation.^10^ Further supporting the views given above, our snRNA‐seq results found that ZBP1 is mainly expressed in macrophages and alters the ratio of M1 and M2 macrophages in LPS‐treated hearts. In addition, macrophages in septic cardiomyopathy should not be viewed only in the context of distinct M1 or M2 polarisation, but also in terms of the continuity of their phenotypes.^22^ In line with their findings, our work demonstrates that deletion of ZBP1 promotes the macrophage polarisation from M1 to M2 by pseudotime analysis. These data underscore a novel mechanism of ZBP1 regulating macrophage activation.

ZBP1 usually triggers inflammatory response or cell death after sensing Z‐form nucleic acids (Z‐NAs).^7^ However, new patterns of ZBP1 activation that independent of Z‐NAs sensing have also been reported, such as the self‐dimerisation of ZBP1 via its RHIM domain.^23^ Regardless of the dependence on Z‐NAs sensing, even by simply increasing the amount of ZBP1, its activation and function will be promoted. Our findings also showed that LPS stimulation upregulates the expression of ZBP1 in heart, while the mechanism underlying the increase of ZBP1 remains a fundamental issue for septic cardiomyopathy. Recently, a variety of transcription factors, such as HSF1,^23^ JNK^24^ and CEBPA,^10^ have been reported to regulate the increased transcription and expression of ZBP1 in different diseases. Yuan et al. reported that heat stress increased HSF1 activation and its binding to the HSF1 binding site within the promoter regions of ZBP1, augmenting the expression and dimerisation of ZBP1 to cause cell death.^23^ In steatotic liver, palmitic acid‐activated JNK signalling activates the transcription of *Zbp1*.^24^ In this investigation, we identified STAT1 as the potential transcription factor of ZBP1. Subsequently, it was verified that STAT1 binds to the *Zbp1* promoter regions and activates *Zbp1* transcription in macrophages. Hozaifa et al. reported that genetic ablation of STAT1 protects against LPS‐induced lethality.^25^ Our findings further demonstrated that the inhibition of STAT1 led to a reduction in LPS‐induced elevated ZBP1 expression. These results complement the transcription mechanism of ZBP1 in sepsis‐induced myocardial dysfunction, and indicate that blockage of STAT1‐ZBP1 axis may be a potential treatment strategy for sepsis and its complications.

In conclusion, this study demonstrates that ZBP1 is activated by LPS to facilitate M1 macrophage polarisation in septic cardiomyopathy, which mediates myocardial dysfunction and inflammatory cell infiltration. These findings establish the potential of macrophage‐derived ZBP1 as a therapeutic target for septic cardiomyopathy and offer a novel mechanism of ZBP1 regulating macrophage activation.

## AUTHOR CONTRIBUTIONS

**Yifan Shi**: Methodology, writing—original draft, investigation. **Lu He**: Investigation. **Jie Ni**: Resources. **Yuyuan Zhou**: Investigation. **Xiaohua Yu**: Investigation. **Yao Du**: Methodology. **Yang Li**: Methodology. **Xi Tan**: Methodology. **Yufang Li**: Investigation. **Xiaoying Xu**: Investigation. **Si Sun**: Writing—review & editing. **Lina Kang**: Resources, supervision. **Biao Xu**: Resources, supervision. **Jibo Han**: Writing—original draft, resources, funding acquisition. **Lintao Wang**: Conceptualisation, methodology, writing—review & editing, funding acquisition.

## CONFLICT OF INTEREST STATEMENT

The authors declare no conflicts of interest.

## ETHICS STATEMENT

All procedures with animals in this study were approved by the Institutional Ethics Committee of Nanjing Drum Tower Hospital and performed in accordance with the guidelines outlined in the Guide for the Care and Use of Laboratory Animals published by the National Institutes of Health (No. 2023AE1072).

## Supporting information



Supporting information

Supporting information

## Data Availability

All data are included within the article or Supplementary Information or available from the authors on request.
