# Supplementary material for: Myeloid deficiency of Z‐DNA binding protein 1 restricts septic cardiomyopathy via promoting macrophage polarisation towards the M2‐subtype
Source: Clin Transl Med. 2025 Apr 27;15(5):e70315. doi: 10.1002/ctm2.70315 (PMC12034574; doi:10.1002/ctm2.70315)
Supplement: Supplementary file 1 — Supporting information [file CTM2-15-e70315-s002.docx]

***Supplementary Materials***

**Myeloid deficiency of ZBP1 restricts septic cardiomyopathy via promoting macrophage polarisation towards the M2-subtype**

**Running title:** Loss of ZBP1 attenuates septic cardiomyopathy.

Yifan Shi^1, *^, Lu He^3, *^, Jie Ni^1, *^, Yuyuan Zhou^4^, Xiaohua Yu^4^, Yao Du^1^, Yang Li^5^, Xi Tan^4^, Yufang Li^1^, Xiaoying Xu^4^, Si Sun^1^, Lina Kang^1,2,4, #^, Biao Xu^1,2,4, #^, Jibo Han^5, #^, Lintao Wang^1,2, #^

* Yifan Shi, Lu He, and Jie Ni contribute equally to this paper.

*^1^ Department of Cardiology, Nanjing Drum Tower Hospital, Affiliated Hospital of Medical School, Nanjing University, Nanjing 210008, Jiangsu, China.*

*^2^Nanjing Key Laboratory for Cardiovascular Information and Health Engineering Medicine, Institute of Clinical Medicine, Nanjing Drum Tower Hospital, Medical School, Nanjing University, Nanjing 210093, Jiangsu, China.*

*^3^ Department of Neurosurgery, The First Affiliated Hospital, Hengyang Medical School, University of South China, Hengyang 421001, Hunan, China.*

*^4^ Department of Cardiology, Nanjing Drum Tower Hospital Clinical College of Nanjing University of Chinese Medicine, Zhongshan Road, Nanjing, 210008, Jiangsu, China.*

*^5^* *Department of Cardiology,* *the Second Affiliated Hospital of Jiaxing University, Jiaxing 314000, Zhejiang, China.*

**Corresponding authors:**

Lintao Wang, Department of Cardiology, Nanjing Drum Tower Hospital, Affiliated Hospital of Medical School, Nanjing University, E-mail: lintaow@126.com.

Jibo Han, Department of Cardiology, The Second Affiliated Hospital of Jiaxing University. Email: jibohanjx2y@163.com.

Biao Xu, Department of Cardiology, Nanjing Drum Tower Hospital, Affiliated Hospital of Medical School, Nanjing University, E-mail: xubiao62@nju.edu.cn.

Lina Kang, Department of Cardiology, Nanjing Drum Tower Hospital, Affiliated Hospital of Medical School, Nanjing University, E-mail: kanglina@njglyy.com.

Supplementary materials contain Supplementary Fig. S1-11.

**
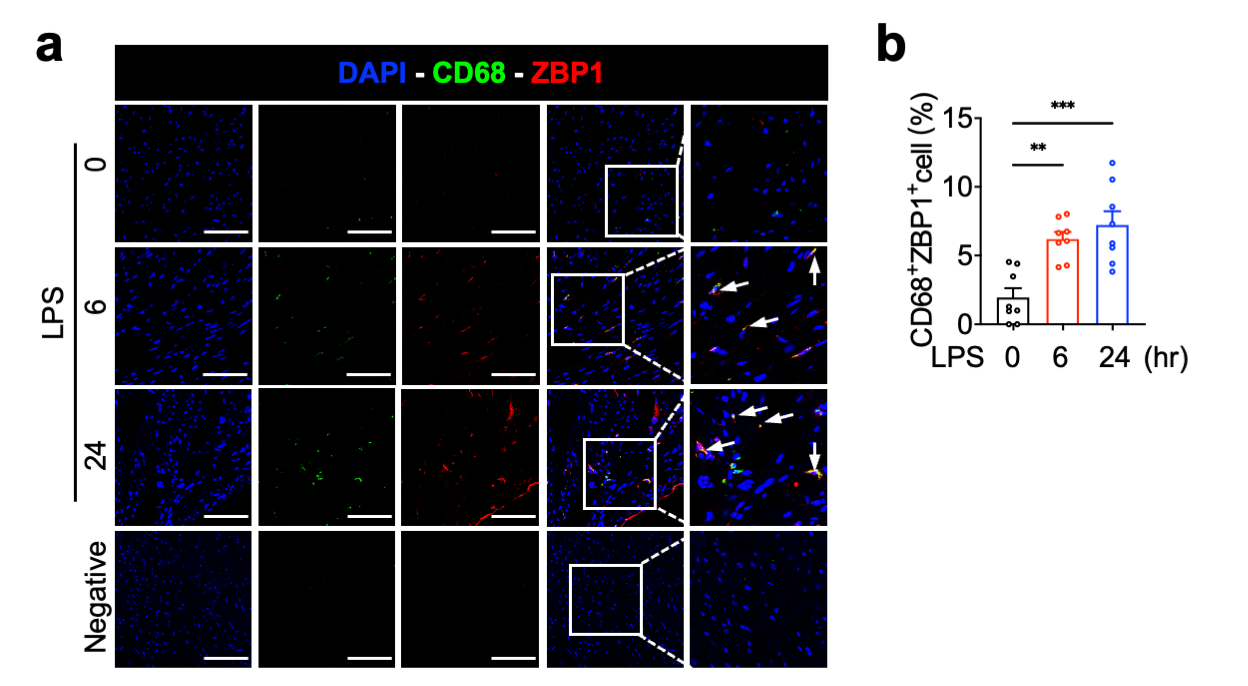
**

**Supplementary Fig. S1 ZBP-positive macrophages were increased after LPS treatment in myocardial tissues**

**(a)** Representative images of the immunofluorescent staining of CD68 and ZBP1 in hearts at course time post intraperitoneal injection of LPS in WT mice (10 mg/kg; 0, 6, or 24 hr). White arrow indicates co-localization of ZBP1 in macrophages. [scale bar = 100 μm, n = 8 in each group]. **(b)** Quantitative analysis of ZBP1^+^ macrophages in hearts at course time post intraperitoneal injection of LPS in WT mice (n=8 in each group).

**
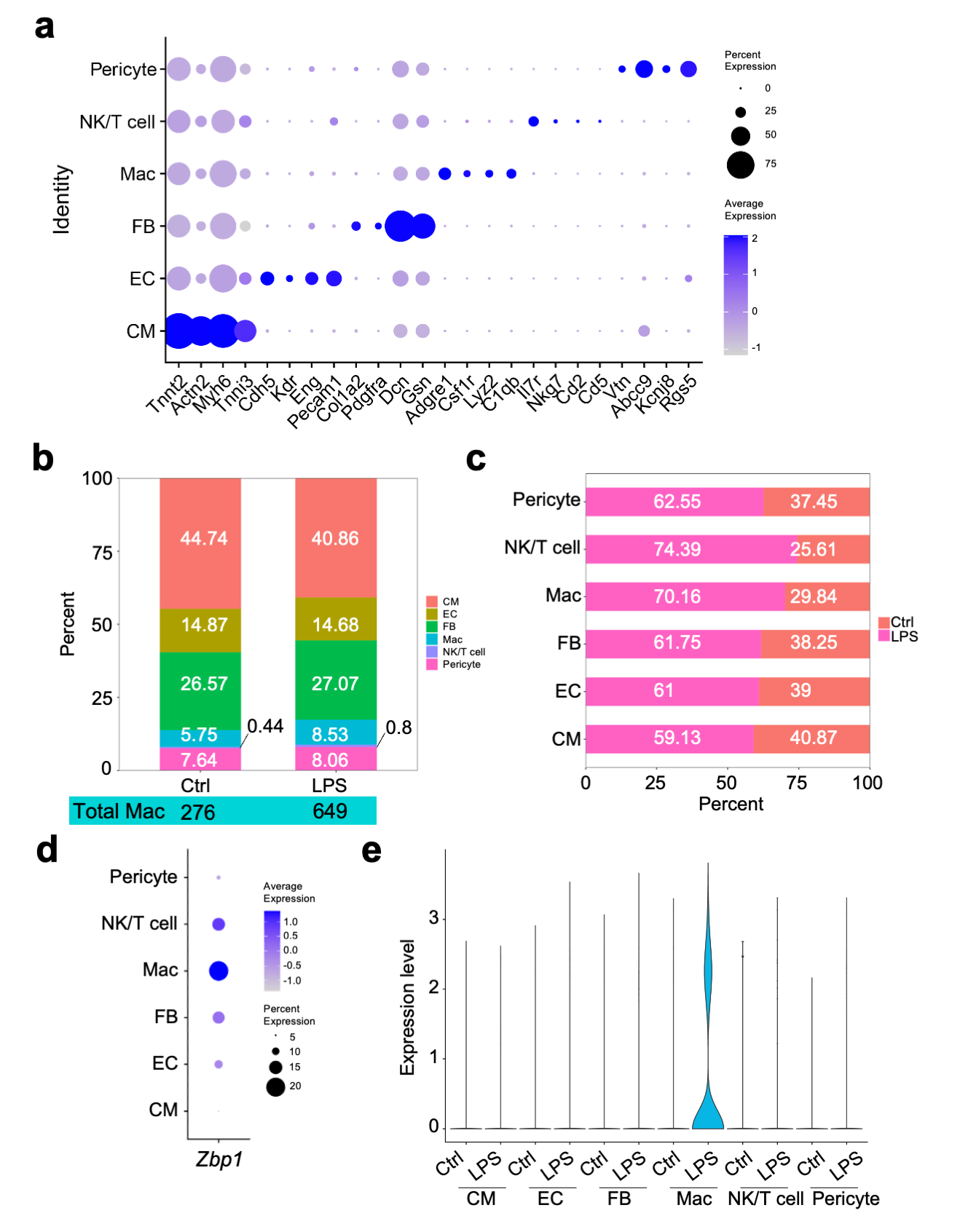
**

**Supplementary Fig. S2 Cell clusters in myocardial tissues by using cell nuclei extraction analysis were determined based on marker genes**

**(a)** Dot plot of known marker genes for each cell cluster identified in **Fig. 1i**. Size of nodes represents percentage of cells expressing a certain gene, and expression scale is shown on the right. **(b)** Bar chart shows the proportions of cell populations in myocardial tissues from Ctrl and LPS mice. Bottom chart shows the numbers of total macrophage. **(c)** Bar chart shows the proportions of different cell types in myocardial tissues from Ctrl and LPS mice. **(d)** Dot plot of *Zbp1* expression in myocardial tissues from both Ctrl and LPS mice. **(e)** Violin plot of *Zbp1* expression in myocardial tissues from Ctrl and LPS mice. CM, cardiomyocyte; EC, endothelial cell; FB, fibroblast; Mac, macrophage.

**
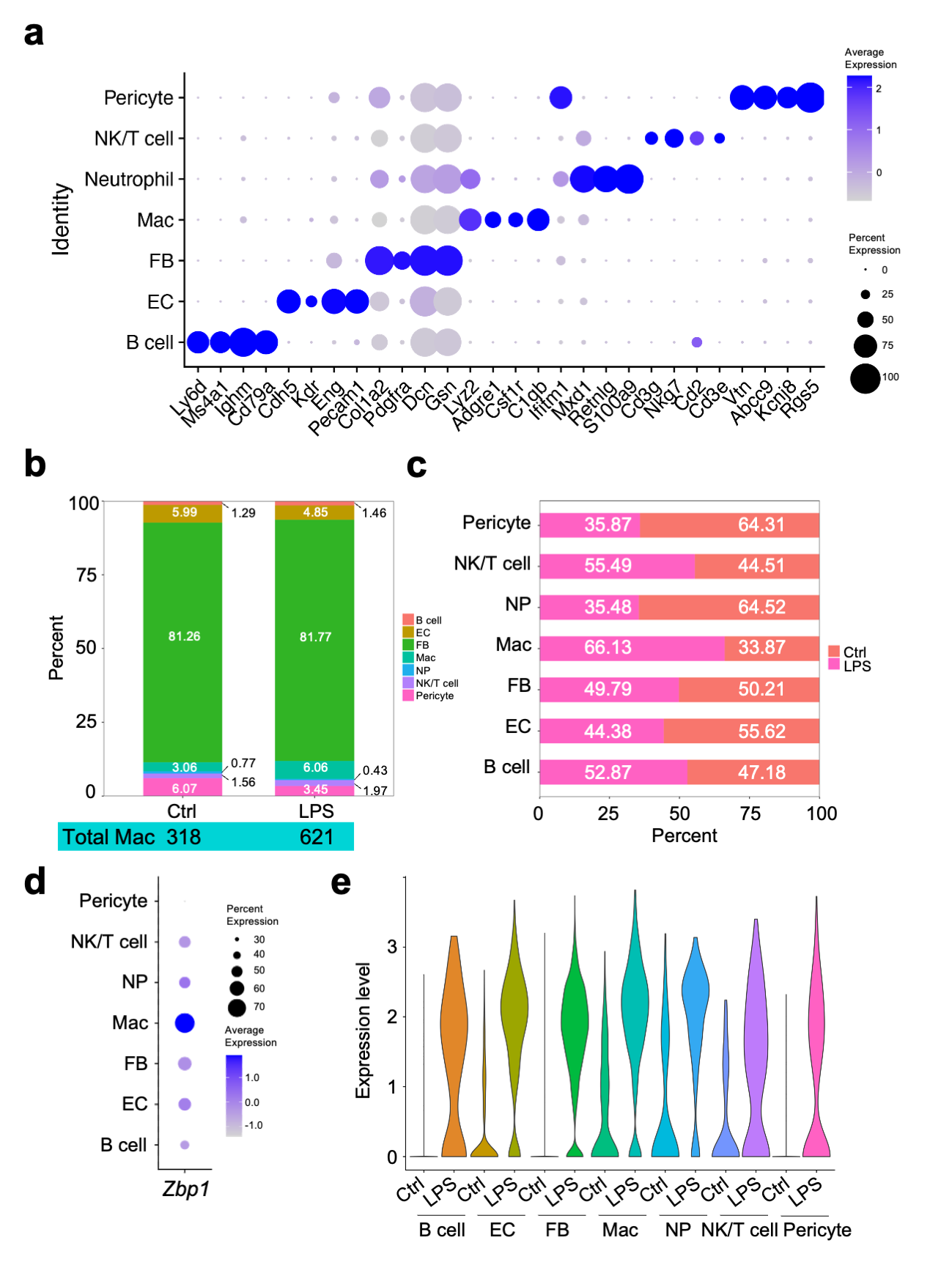
**

**Supplementary Fig. S3 Cell clusters in myocardial tissues by using** **cell suspension analysis were determined based on marker genes**

**(a)** Dot plot of known marker genes for each cell cluster identified in **Fig. 1l**. Size of nodes represents percentage of cells expressing a certain gene, and expression scale is shown on the right. **(b)** Bar chart shows the proportions of cell populations in myocardial tissues from Ctrl and LPS mice. Bottom chart shows the numbers of total macrophage. **(c)** Bar chart shows the proportions of different cell types in myocardial tissues from Ctrl and LPS mice. **(d)** Dot plot of *Zbp1* expression in myocardial tissues from both Ctrl and LPS mice. **(e)** Violin plot of *Zbp1* expression in myocardial tissues from Ctrl and LPS mice. EC, endothelial cell; FB, fibroblast; Mac, macrophage; NP, neutrophil.

**
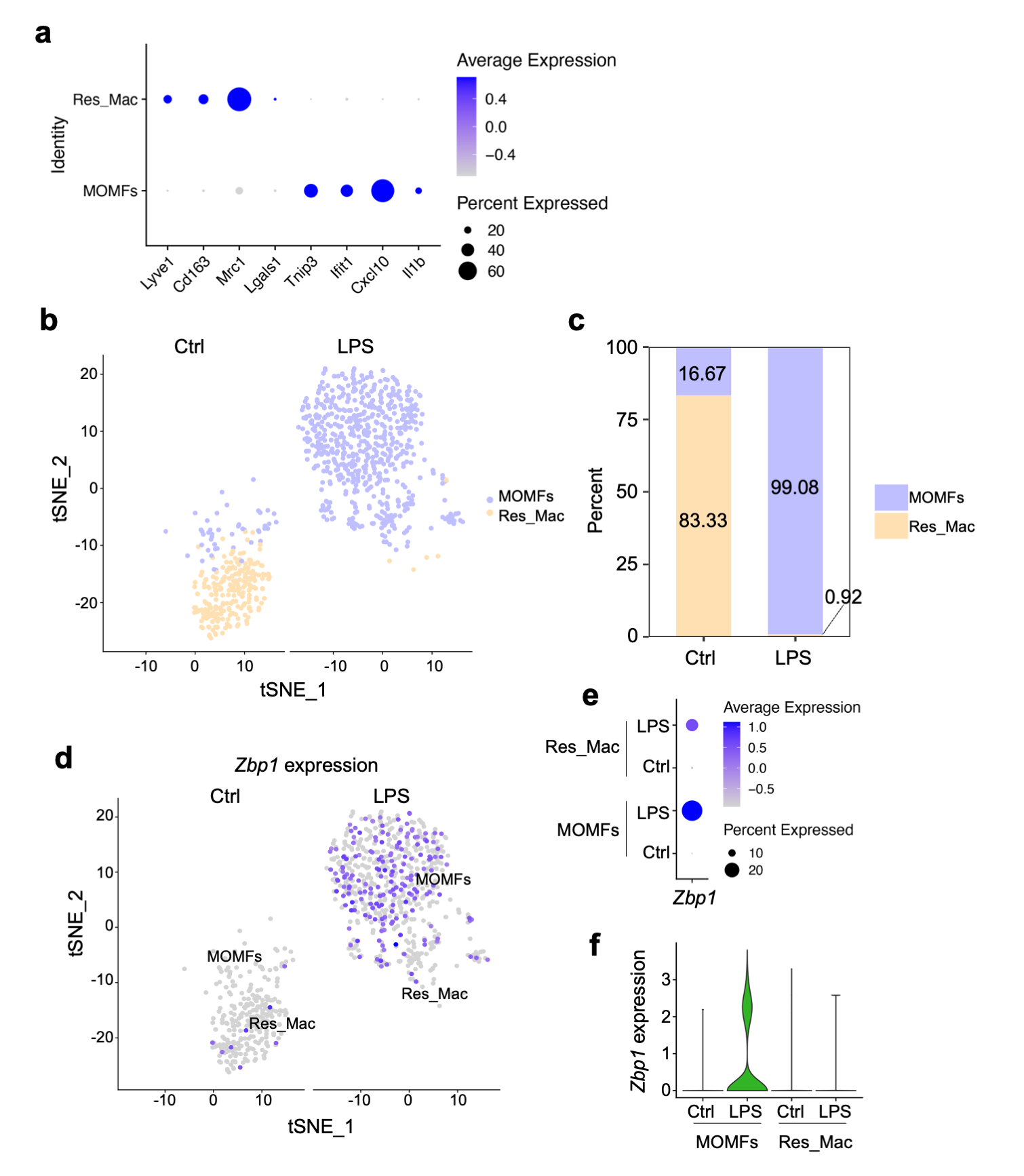
**

**Supplementary Fig. S4 *Zbp1* expression in cardiac resident macrophages (Res_Mac) and infiltrating monocyte-derived macrophages (MOMFs) by using cell nuclei extraction analysis**

**(a)** Dot plot of known marker genes for cardiac Res_Mac and MOMFs. Size of nodes represents percentage of cells expressing a certain gene, and expression scale is shown on the right. **(b)** tSNE plot of cell clusters in myocardial tissues from Ctrl and LPS mice by using cell nuclei extraction analysis. **(c)** Bar chart shows the proportions of Res_Mac and MOMFs in myocardial tissues from Ctrl and LPS mice. **(d-f)** tSNE plot **(d)**, Dot plot **(e)**, and Violin plot **(f)** of *Zbp1* expression in cardiac Res_Mac and MOMFs from both Ctrl and LPS mice.

**
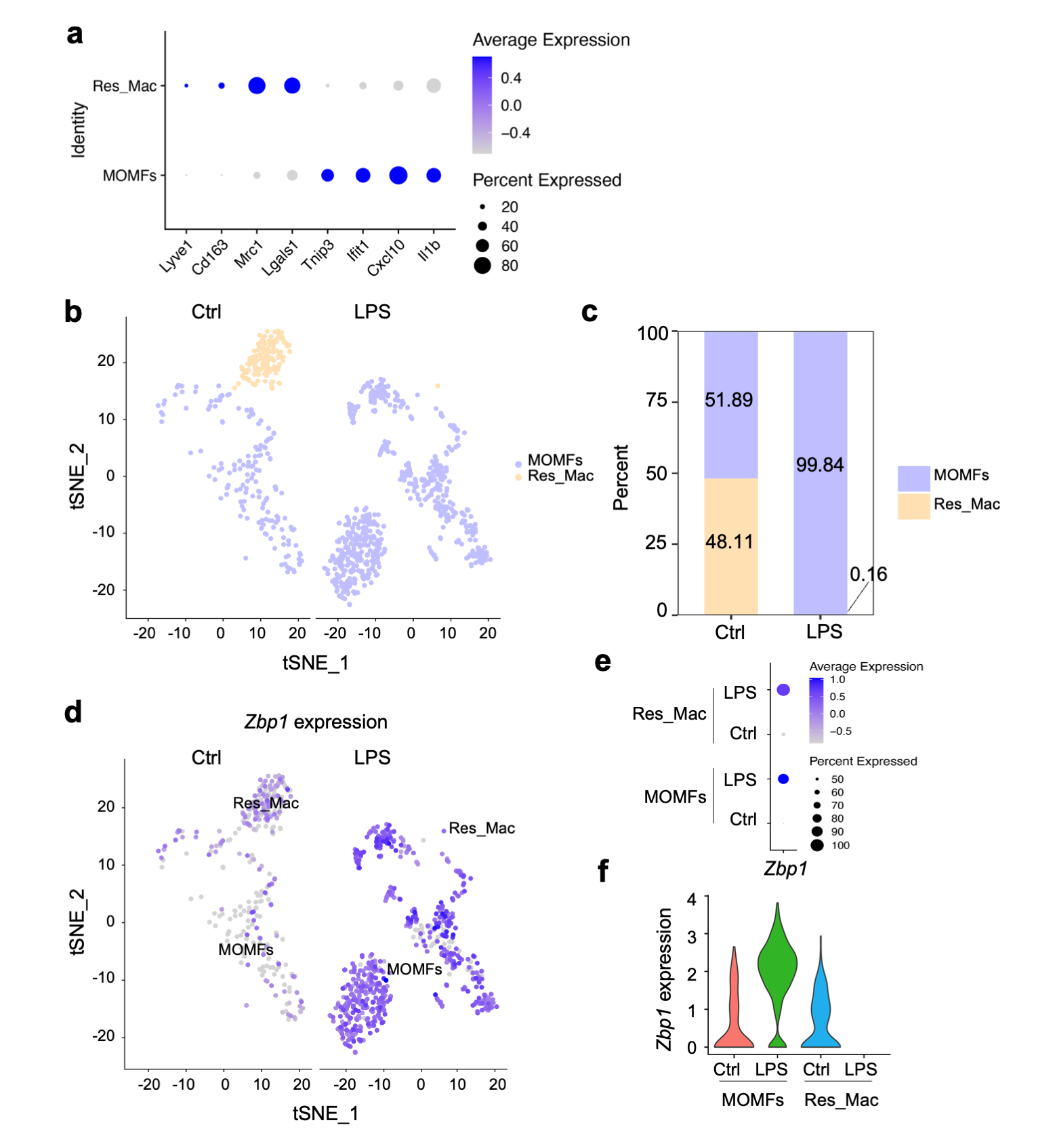
**

**Supplementary Fig. S5 *Zbp1* expression in cardiac resident macrophages (Res_Mac) and infiltrating monocyte-derived macrophages (MOMFs) by using** **cell suspension analysis**

**(a)** Dot plot of known marker genes for cardiac Res_Mac and MOMFs. Size of nodes represents percentage of cells expressing a certain gene, and expression scale is shown on the right. **(b)** tSNE plot of cell clusters in myocardial tissues from Ctrl and LPS mice by using cell suspension analysis. **(c)** Bar chart shows the proportions of Res_Mac and MOMFs in myocardial tissues from Ctrl and LPS mice. **(d-f)** tSNE plot **(d)**, Dot plot **(e)**, and Violin plot **(f)** of *Zbp1* expression in cardiac Res_Mac and MOMFs from both Ctrl and LPS mice.

**
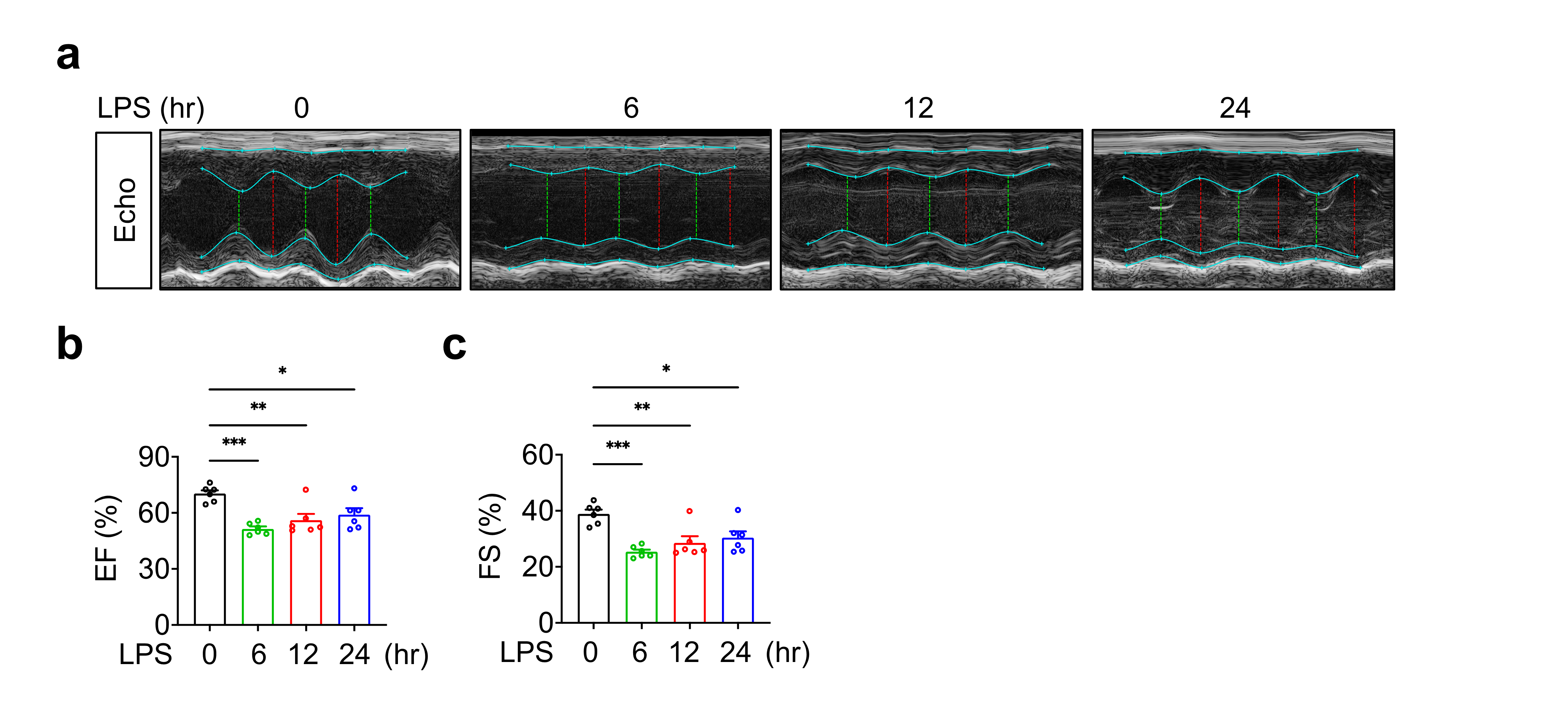
**

**Supplementary Fig. S6 The effect of LPS time gradient on cardiac function in WT mice**

**(a)** Representative echocardiography M-mode images obtained from mice at course time (0, 6, 12, and 24 hr) after LPS administration (10 mg/kg). **(b-c)** Left ventricular ejection fraction (EF) and left ventricular fraction shortening (FS) were quantified via echocardiography (n=6 in each group).

Mean ± SEM; *P<0.05, **P<0.01, ***P<0.001.

**
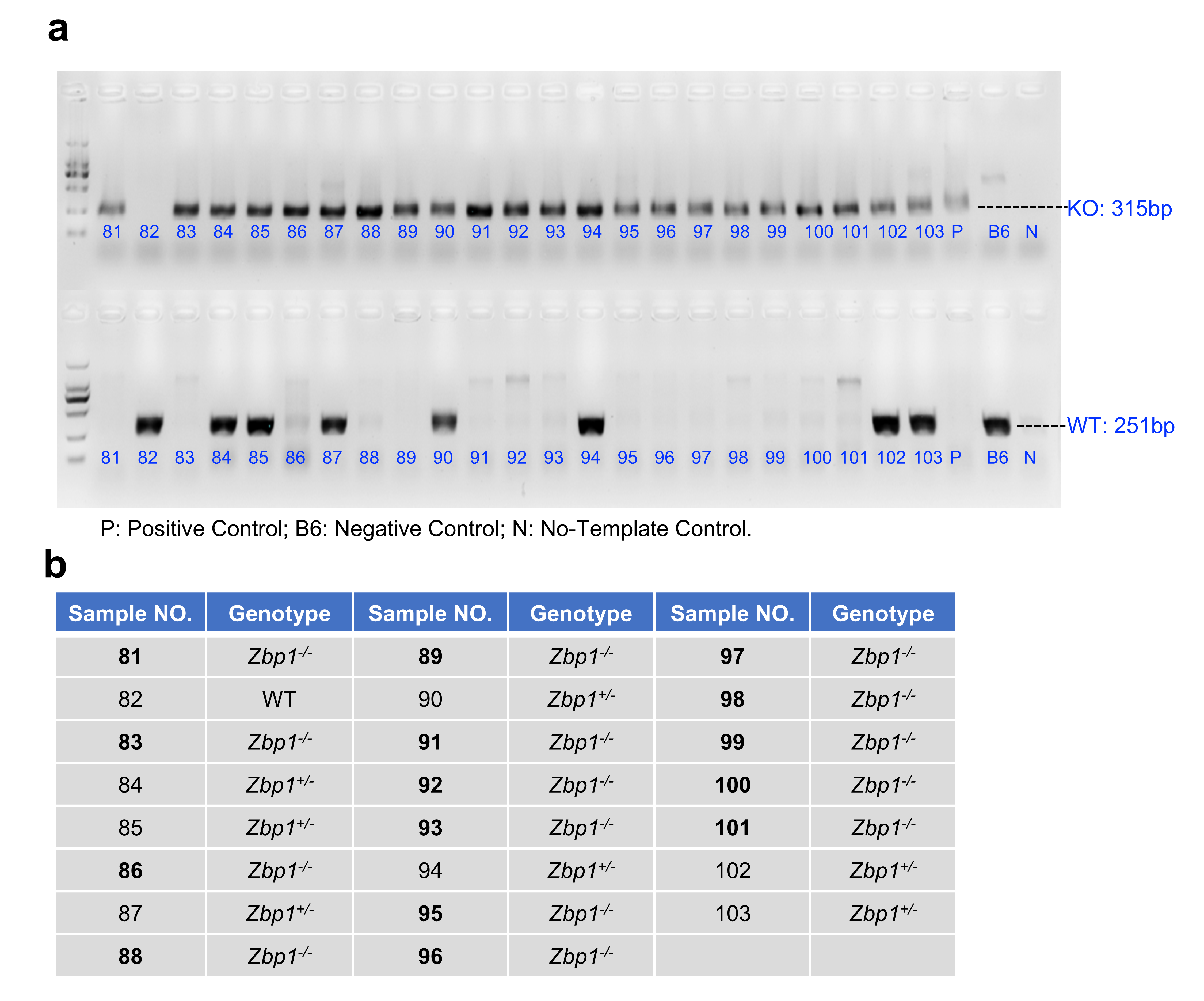
**

**Supplementary Fig. S7 Genotyping of *Zbp1^-/-^* mice**

**(a-b)** DNA exacted from mouse tails performs PCR amplification using primers of KO (WT: 12128 bp; KO: 315 bp) and WT (WT: 251 bp; KO: 0 bp). Gel electrophoresis shows the genotype of WT, *Zbp1^-/-^* and *Zbp1^+/-^* mice (P: Positive control; B6: Negative control; N: No-template control).

**
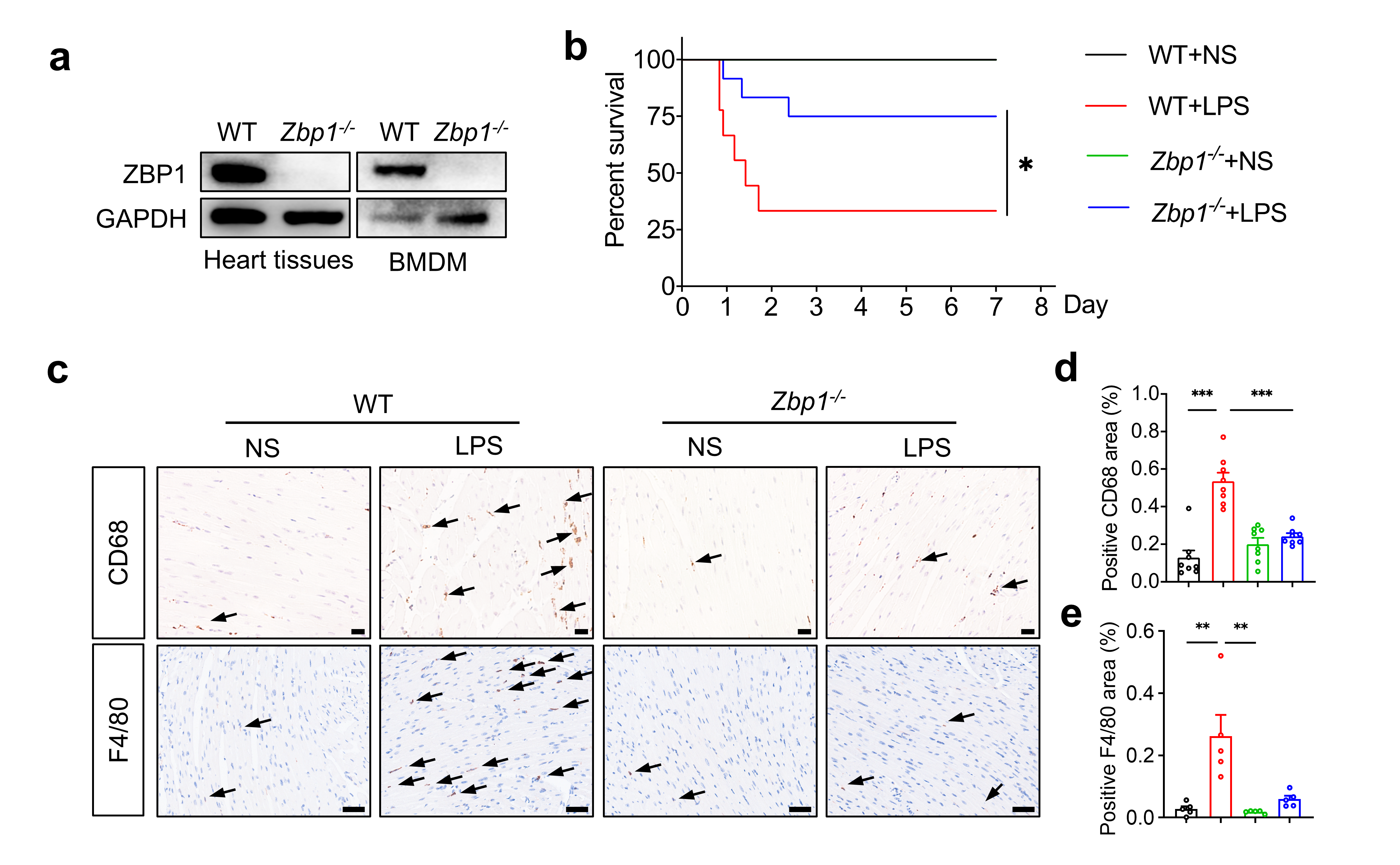
**

**Supplementary Fig. S8 Deletion of ZBP1 improved the survival of septic mice**

**(a)** *Zbp1* gene knockout efficiency in hearts and BMDMs was detected by Western blot. **(b)** Survival of WT and *Zbp1^-/-^* mice intraperitoneally injected with saline or LPS, monitored for a 7-day period (n = 9 for WT+NS, WT+LPS, and *Zbp1^-/-^*+NS; n = 12 for *Zbp1^-/-^*+LPS). Log-rank (Mantel-Cox) test (conservative) was used for comparison of survival curves. (c) Representative images of immunohistochemistry staining of CD68 and F4/80 at 6 hr after intraperitoneal injection of saline or LPS in WT or Zbp1^-/-^ mice. Black arrow indicates positive area of CD68 and F4/80. [scale bar = 20 μm and 50 μm]. (d) Quantitative analysis of CD68^+^ area in myocardial tissues (n = 8 in each group). (e) Quantitative analysis of F4/80^+^ area in myocardial tissues (n = 5 in each group).

Mean ± SEM; *P<0.05, **P<0.01, ***P<0.001.

**
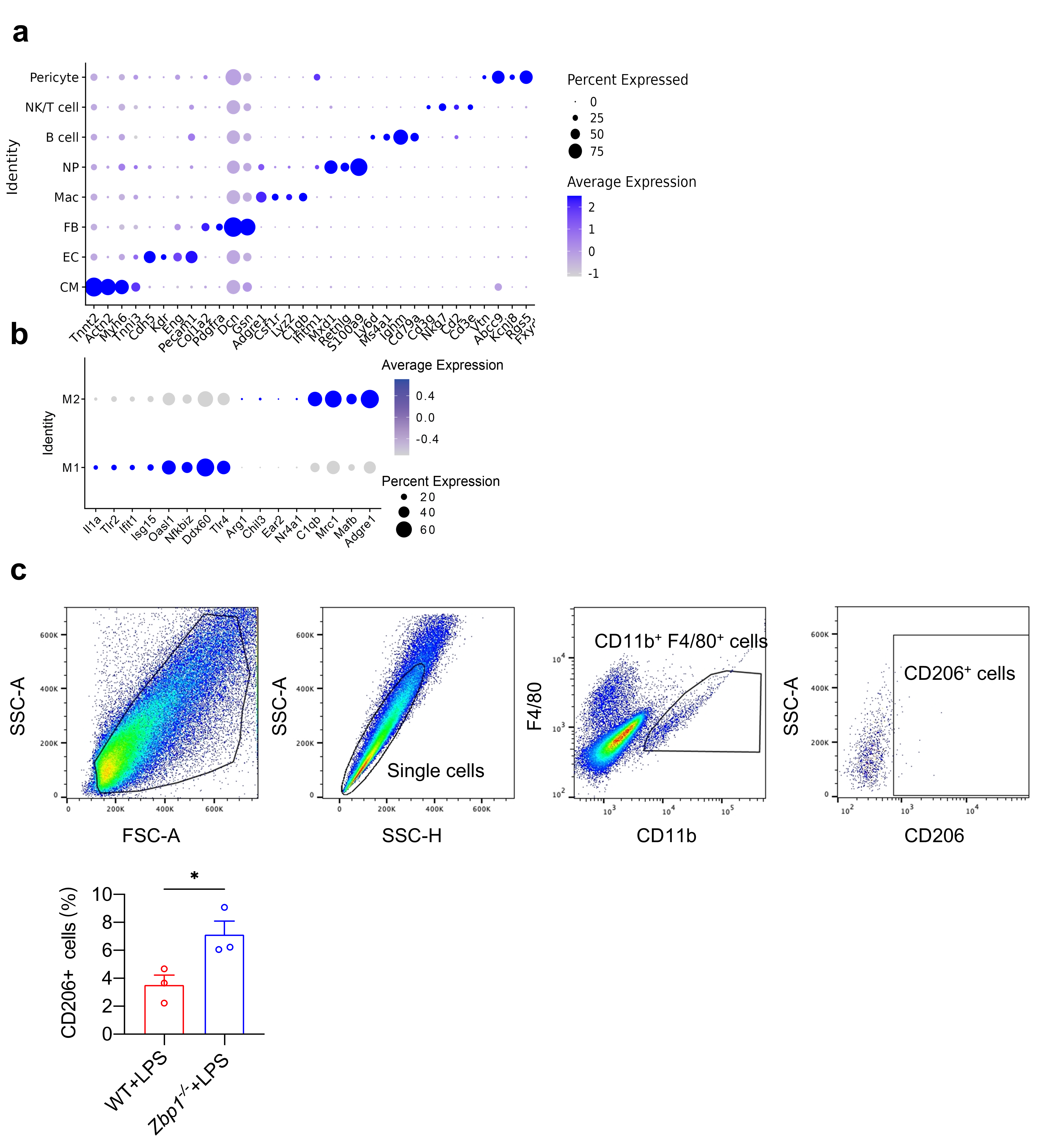
**

**Supplementary Fig. S9 Cell clusters in myocardial tissues were determined based on marker genes**

**(a)** Dot plot of known marker genes for each cell cluster identified in **Fig. 3a**. **(b)** Dot plot of known marker genes for M1 and M2 macrophage clusters identified in **Fig. 3d**. Size of nodes represents percentage of cells expressing a certain gene, and expression scale is shown on the right. **(c)** Flow cytometric analysis of the heart further validated the ratio of CD206^+^ CD11b^+^ F4/80^+^ cells (M2 macrophage) in LPS mice (n=3 in each group).

Mean ± SEM; *P<0.05.

**
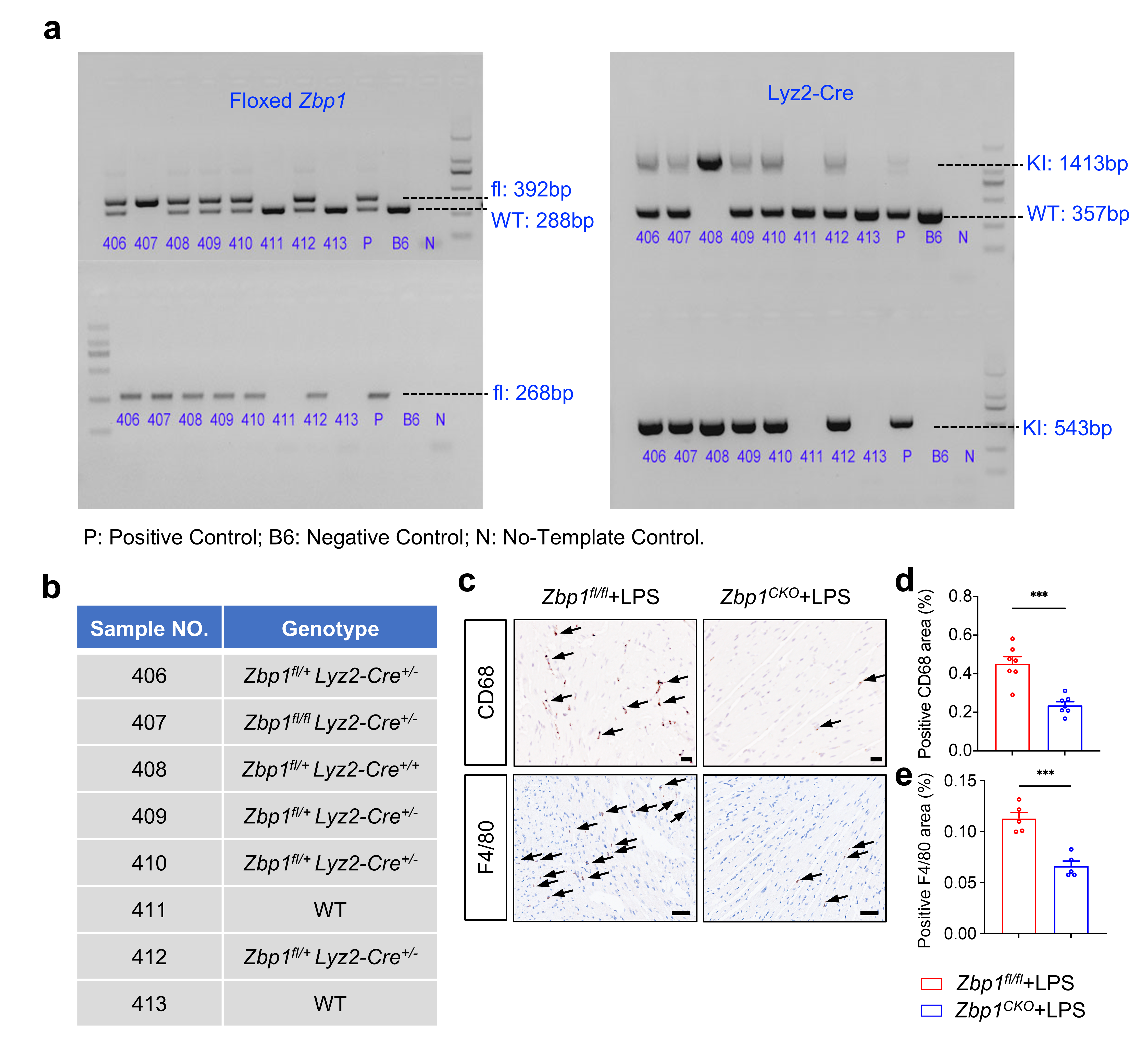
**

**Supplementary Fig. S10 Genotyping of *Zbp1^fl/fl^* and *Zbp1^CKO^* mice**

**(a-b)** DNA exacted from mouse tails performs PCR amplification using primers of WT, Flox and Cre. Gel electrophoresis shows the genotype of *Zbp1^fl/+^ Lyz2-Cre^+/-^*, *Zbp1^fl/fl^ Lyz2-Cre^+/-^*, *Zbp1^fl/+^ Lyz2-Cre^+/+^*, and WT mice (P: Positive control; B6: Negative control; N: No-template control). (c) Representative images of immunohistochemistry staining of CD68 and F4/80 at 6 hr after intraperitoneal injection of LPS in *Zbp1^fl/fl^* or *Zbp1^cko^* mice. [scale bar = 20 μm and 50 μm]. (d) Quantitative analysis of CD68^+^ area in myocardial tissues (n = 7 in each group). (e) Quantitative analysis of F4/80^+^ area in myocardial tissues (n = 5 in each group).

Mean ± SEM; ***P<0.001.


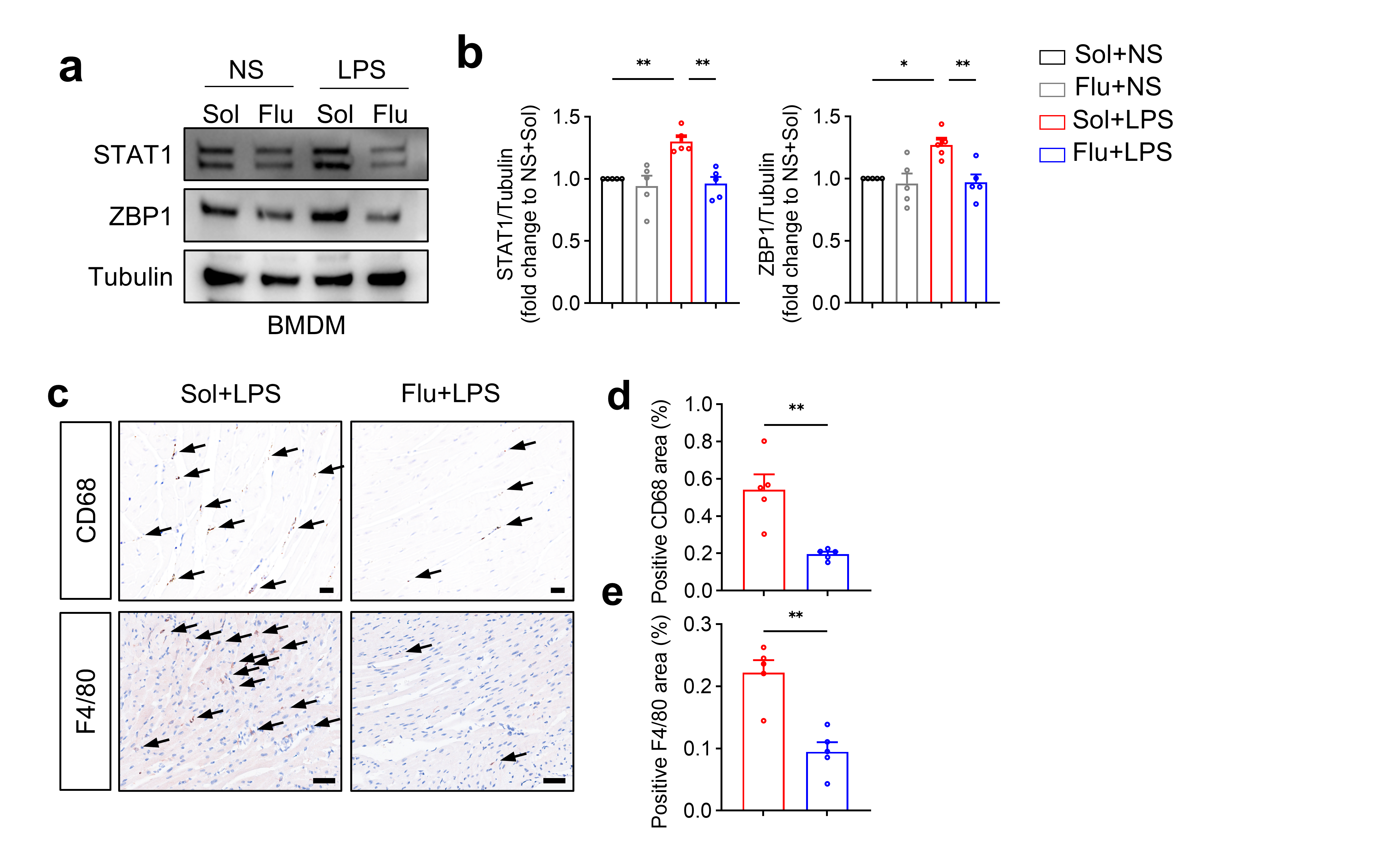


**Supplementary Fig. S11 Fludarabine inhibits LPS-induced expression of STAT1 and ZBP1 *in vitro***

(a-b) BMDM was pretreated with Fludarabine (100 μM) or solvent for 1 h, followed by treatment with LPS (10 μg/mL) or NS for 6 h. Western blot analyzes the protein level of STAT1 and ZBP1 in BMDM (n = 5 independent experiments). (c) Representative images of immunohistochemistry staining of CD68 and F4/80 at 6 hr after intraperitoneal injection of LPS in solvent or fludarabine-treated mice. [scale bar = 20 μm and 50 μm]. (d) Quantitative analysis of CD68^+^ area in myocardial tissues (n = 5 in each group). (e) Quantitative analysis of F4/80^+^ area in myocardial tissues (n = 5 in each group).

Mean ± SEM; *P<0.05, **P<0.01.
