# Supplementary material for: Myeloid deficiency of Z‐DNA binding protein 1 restricts septic cardiomyopathy via promoting macrophage polarisation towards the M2‐subtype
Source: Clin Transl Med. 2025 Apr 27;15(5):e70315. doi: 10.1002/ctm2.70315 (PMC12034574; doi:10.1002/ctm2.70315)
Supplement: Supplementary file 2 — Supporting information [file CTM2-15-e70315-s001.docx]

**Supplementary Table 1: Primer sequences for genotyping.**

| Gene | Primer sequence (5’-3’) | Note |
| --- | --- | --- |
| *Zbp1* | F1: GGAGGATTGCTATGAGTTCCAGG  R1: CTCTGGGTAGCTGATTCTTCCTCT | WT band: 12128 bp  Mutant band: 315 bp |
|  | F2: AAAGATCCTGCAGGTGTTGAGC  R2: GTCTGCTCTGCCTACATGACAGATT | WT band: 251 bp |
| *Floxed Zbp1* | F1: GGAGGATTGCTATGAGTTCCAGG  R1: CCTGATACAGCAGGAGTCCTGAA | WT: 357 bp  Mutant band: 1413 bp |
|  | F2: TCTGAGGCGGAAAGAACCAG  R2: CTCTGGGTAGCTGATTCTTCCTCT | Mutant band: 543 bp |
| *Lyz2-Cre* | F1: AGTGCTGAAGTCCATAGATCGG  R1: GTCACTCACTGCTCCCCTGT | WT: 357 bp  Cre band: 1413 bp |
|  | F2: AGTGCTGAAGTCCATAGATCGG  R2: CTGATTCTCCTCATCACCAGG | Cre band: 543 bp |

Annotations: F, forward primer; R, reverse primer.

**Supplementary Table 2: Predicated TFs from PROMO database.**

| **Predicated TFs from PROMO database** | | | |
| --- | --- | --- | --- |
| YI | c-Fos | COE1 | Pax-6 |
| TCF-1(P) | MyoD | GR | POU2F1b |
| HNF-3beta | NF-AT4 | STAT1 | E2F-1 |
| RXR-alpha | YY1 | c-Rel | AP-1 |
| GATA-2 | POU2F2 | POU2F1a | NF-kappaB |
| GATA-1 | NF-AT1 | POU5F1 | Nkx-1 |
| CP2 | CRE-BP2 | DEC2 | F(alpha)-f |
| myogenin | Sp1 | JunD | POU2F1c |
| HES-1 | HOXA5 | LyF-1 |  |
| HNF-3 | NF-1 | USF-1 |  |
| TFE3-S | Cebpa | AhR |  |
| Pax-5 | Spi1 | Cebpb |  |
| Tal-1 | Rela | c-Jun |  |
| HNF-6 | TCF-2 | MTF-1 |  |

Annotations: TFs, transcription factor.

**Supplementary Table 3: Reported TFs in BMDM from CHIP atlas.**

| **Reported TFs in BMDM from CHIP atlas** | | | |
| --- | --- | --- | --- |
| CEBPB | ATF3 | IRF1 | Smarca4 |
| CTCF | CEBPD | STAT6 | Fli1 |
| JUN | STAT1 | IRF9 | IRF8 |
| Rela | JUND | ELF4 | Nr1h2 |
| Spi1 | JUNB | IRF3 | FOS |
| Rad21 | ADNP | ELF1 | Egr2 |
| Creb1 | Srebf1 | STAT2 | Nr3c1 |
| Ep300 | USF2 | BRD4 |  |

Annotations: TFs, transcription factor.

**Supplementary Table 4: Predicated TFs in pySCENIC of snRNA-seq.**

| **Regulon** | **Gene** |
| --- | --- |
| CEBPB | Zbp1 |
| EVT6 | Zbp1 |
| IRF2 | Zbp1 |
| PM1 | Zbp1 |
| STAT1 | Zbp1 |
| STAT2 | Zbp1 |

Annotations: TFs, transcription factor.
